
JOURNAL INFORMATION
==============================
NLM Title Abbreviation: Insights Imaging
Journal ID: Insights Imaging
NLM Journal ID: 101532453

Insights into Imaging

EISSN: 1869-4101
Publisher: Springer

ARTICLE INFORMATION
==============================
PMCID: PMC9532479
PMID: 36195808
DOI: 10.1186/s13244-022-01285-6
Article ID: 1285
Article version: 1
Subjects: Meeting Abstracts

ESHNR 2022 Book of Abstracts

Electronic publication date: 2022 Oct 5
Publication date: 2022 Oct
Volume: 13
Issue: Suppl 3
Issue sponsor: Publication costs were funded by the European Society of Head and Neck Radiology.
Electronic Location ID: 157
Copyright: © The Author(s) 2022
License: Open AccessThis article is licensed under a Creative Commons Attribution 4.0 International License, which permits use, sharing, adaptation, distribution and reproduction in any medium or format, as long as you give appropriate credit to the original author(s) and the source, provide a link to the Creative Commons licence, and indicate if changes were made. The images or other third party material in this article are included in the article's Creative Commons licence, unless indicated otherwise in a credit line to the material. If material is not included in the article's Creative Commons licence and your intended use is not permitted by statutory regulation or exceeds the permitted use, you will need to obtain permission directly from the copyright holder. To view a copy of this licence, visit http://creativecommons.org/licenses/by/4.0/.
License URL: https://creativecommons.org/licenses/by/4.0/

Conference name: ESHNR 2022
Conference location: Virtual
Conference date: 6-8 October 2022
issue-copyright-statement: © The Author(s) 2022

==============================

eshnr
european society of
head and neck radiology

### Insights into Imaging

Education and strategies in European radiology

ESHNR 2022 Book of Abstracts / Volume 13 / Supplement 3 / October 2022

ESHNR 2022 / October 6–8 / Online Edition
34th Annual Meeting and Refresher Course

ESR European Society of Radiology eshnr european society of
head and neck radiology

### ESHNR Masterclass 2022

**December 08-10, 2022 online**

Topic: Soft Tissue Neck/Upper Aerodigestive Tract

**Register now!**

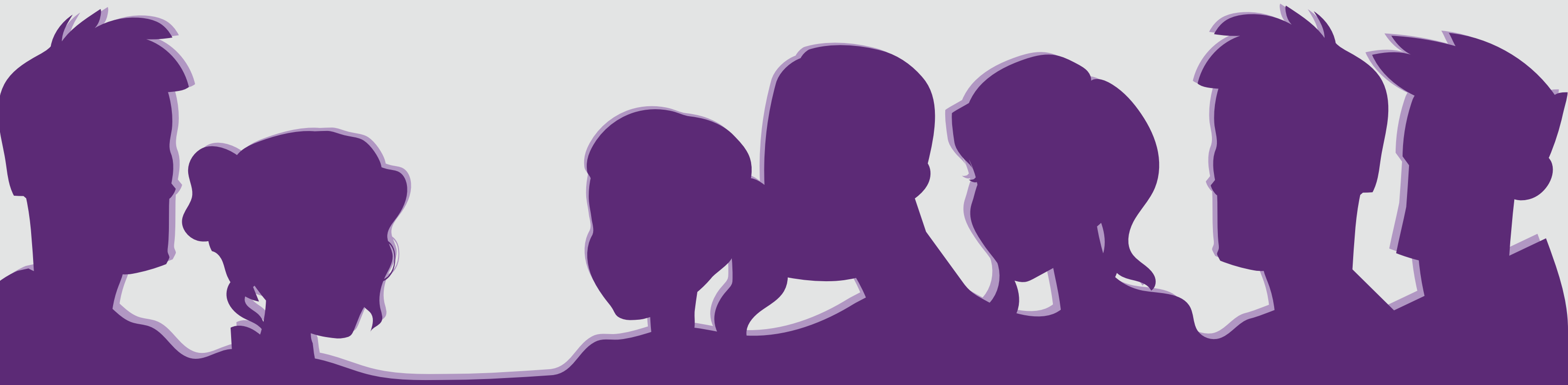

www.eshnr.eu

### Congress Programme

The Scientific Programme will offer a wide spectrum of lectures covering the scope of head and neck radiology, including spatial anatomy, cancer imaging, the orbit, sinonasal and nasopharyngeal region, temporal bone, larynx and hypopharynx, thyroid, parathyroid and salivary glands, led by distinguished speakers from our society and worldwide. As always, there will be sessions dedicated to scientific presentations where you can familiarize yourself with the latest developments in head and neck radiology.

This year there will be a special focus on dentomaxillofacial imaging with a whole day of parallel sessions dedicated to this topic. Together with the Baltic Congress of Radiology and guest speakers from the ASHNR we will have joint highlight sessions on emergency imaging and guidelines in H&N radiology. As a novelty, a special session on education in head and neck radiology, will be aimed at our junior colleagues with deeper interest in the field. Our collaboration session with the ESNR will have its focus on meningeal diseases.

### Organising Secretariat

Central ESHNR Office
Am Gestade 1
1010 Vienna
office@eshnr.eu | www.eshnr.eu

### Confirmation of Attendance

Each participant who successfully registered and attended the live event will receive a confirmation of attendance after the meeting.

### Disclaimer

The ESHNR 2022 Book of Abstracts summarises the presentations accepted to be held at the Annual Scientific Meeting - Online Edition | October 2022. Abstracts were submitted by the authors warranting that good scientific practice, copyrights and data privacy regulations have been observed and relevant conflicts of interest declared. Abstracts reflect the authors' opinions and knowledge. The ESHNR does not give any warranty about the accuracy or completeness of medical procedures, diagnostic procedures or treatments contained in the material included in this publication. The views and opinions presented in all given abstracts and presentations, including scientific, educational and professional matters, do not necessarily reflect the views and opinions of the ESHNR. In no event will the ESHNR be liable for any direct or indirect, special, incidental, consequential, punitive or exemplary damages arising from the use of these abstracts.

In preparing this publication, every effort has been made to provide the most current, accurate, and clearly expressed information possible. Nevertheless, inadvertent errors in information can occur. The ESHNR is not responsible for typographical errors, accuracy, completeness or timeliness of the information contained in this publication

Insights Imaging (2022) 13 (Suppl 3): 157
https://doi.org/10.1186/s13244-022-01285-6
Published: 05 October 2022

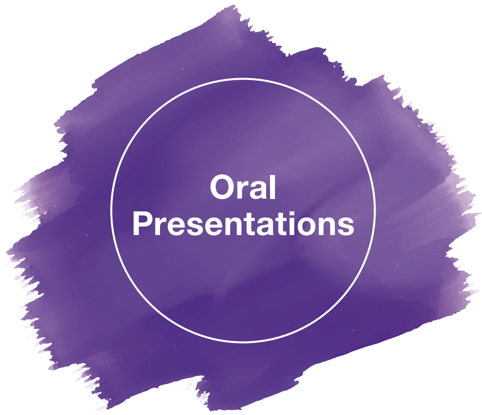

### Radiomics feature analysis for early HPV status determination in oropharyngeal cancer: An interim analysis

*Authors: C. Giannitto[1, 2], G. Carnicelli[1, 2], S. Lusi[1, 2], L. Manganaro[3], E. Casiraghi[4], G. Mercante[5], G. Spriano[5], M. Scorsetti[6], M. Francone[1, 2], S. L. Politi[1, 2], L. Balzarini[1]; [1]Humanitas Clinical and Research Center IRCCS, Radiology Department, Rozzano, Italy, [2]Humanitas University, Pieve Emanuele, Department of Biomedical Sciences, Rozzano, Italy, [3]Policlinico Umberto I, Sapienza University of Rome, Department of Radiological, Oncological and Pathological Sciences, Rome, Italy, [4]University of Milan, Department of Computer Science, Milan, Italy, [5]Humanitas Clinical and Research Center IRCCS, Otolaryngology Unit, Rozzano, Italy, [6]Humanitas Clinical and Research Center IRCCS, Radiotherapy Unit, Rozzano, Italy*

**Purpose/Objectives:** Human Papillomavirus (HPV) status is a key determinant of oropharyngeal squamous cell cancer (OPSCC) management and prognosis. We aimed at building a magnetic resonance imaging(MRI)-based radiomics signature predictive of HPV-status, additionally inferring associations with “lymph node positivity” and “recurrence”

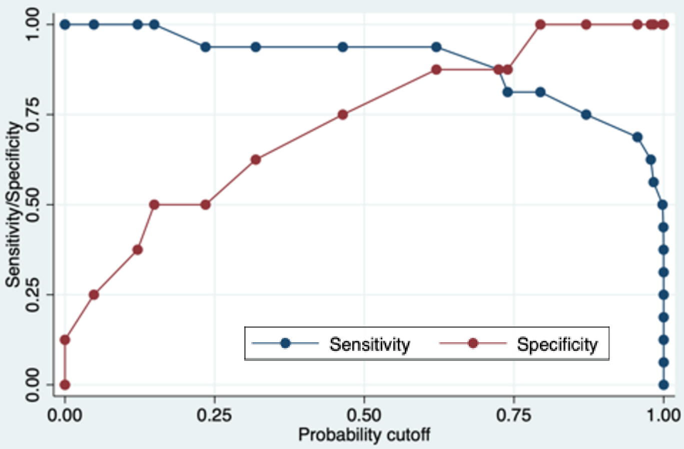

Sensitivity and Specificity analysis for the T2WI-based model, trained for HPV status

**Methods and materials:** A total of 102 treatment-naïve patients with histopathologic diagnosis of OPSCC were enrolled. We extracted radiomics features from Axial spin-echo T2-weighted (T2WI) and 3D GE fat-suppressed T1-weighted sequence (3D-GE-T1) after contrast medium injection. HPV-DNA assessment was performed for all participants. 224 features were extracted for each patient using 3D-Slicer v.4.11 software; pre-processing involved voxel resampling, inhomogeneity correction (N4ITK algorithm) and intensity normalization. Mann-Whitney U, Spearman’s rho non-parametric tests were performed for feature selection and to infer strength of association with with clinical outcomes of interest (lymph node status, stage, extrinsic muscle involvement, recurrence). Radiomics-based algorithms generated by binary logistic regression were trained for the outcome “HPV status”, “lymph node positivity”, “disease recurrence”. Postestimation analysis involved estimation of sensitivity, specificity and diagnostic accuracy expressed as area under the curve (AUC) of receiver operating curves.

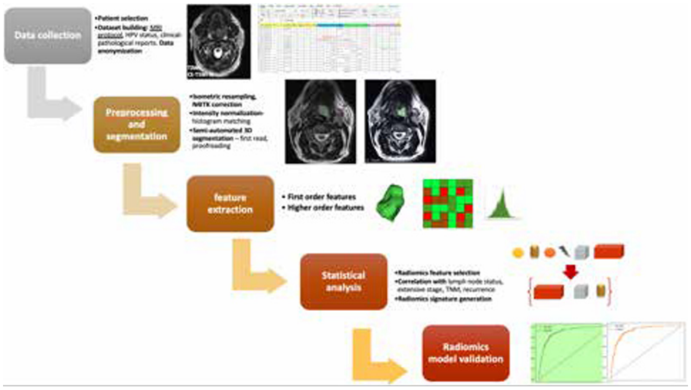

Workflow of the study, from patient selection to image acquisition and analysis

**Result:** Seven T2WI-derived features resulted significantly predictive of HPV status among kurtosis (r=0.41; p 0.047), entropy (r=0.504; p 0.012), Gray Level Dependence Matrix features such as emphasis (p 0.04); two derived features extracted from 3D-GE-T1 ( sphericity and surface volume ratio) revealed significant predictivity (p=0.025). Specificity of the T2WI classifier was 87.5 %, sensitivity 93.7 %, with AUC=0.953; performance increased with combined T2 and 3D-GE-T1 features. Correlation analysis showed significant predictivity of T2WI features for lymph node involvement: energy (p 0.04), entropy (p 0.02), autocorrelation and grey-level co-occurrence matrix features were the most representative. Spearman’s correlation revealed significant associations of T2WI and 3D-GE-T1-derived features with lymph node status, stage, and recurrence.

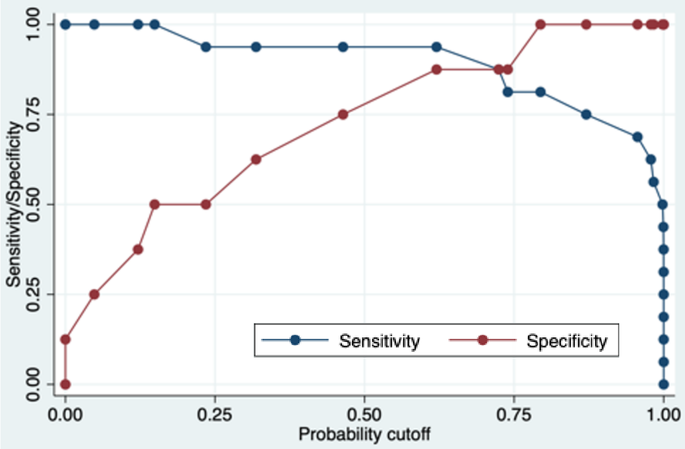

Sensitivity and Specificity analysis for the T2WI-based model, trained for HPV status

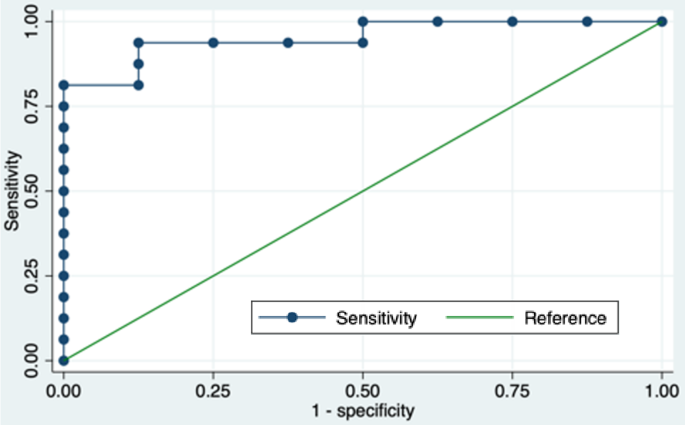

Receiver Operating Curve with relative Area Under the Curve for the T2WI-based algorithm trained for HPV-status

**Conclusion:** Radiomics based algorithms could be accurate noninvasive predictors of HPV status, lymph node positivity, and disease recurrence. Our preliminary results will be validated on a larger cohort.

### AI-Based Automatic Segmentation of Craniomaxillofacial Anatomy from CBCT Scans for Automatic Detection of Pharyngeal Airway Evaluations in OSA patients

*Authors:* *K. Orhan[1], M. Shamshiev[2], M. Ezhov[2], A. Plaskin[2], A. Kurbanova[3], G. Unsal[3], M. Gurasev[2], M. Golitsyna[2], S. Aksoy[3], M. Mısırlı[3], F. Rasmussen[4], E. Shumilov[2], A. Sanders[2]; [1]Ankara University, Ankara, Turkey, [2]Diagnocat Inc, San Francisco, United States, [3]Near East University, Nicosia, Cyprus, [4]SVS Esbjerg, Internal Medicine Department Lunge Section, Esbjerg, Denmark*

**Purpose/Objectives:** Numerous softwares are available in order to analyze the CBCT data with semi-automatic or manual volumetric measurement process. However most of those softwares are laborious, time-consuming and a completely automatic airway detection algorithm is limited. Thus, the aim of this is to generate and also validate an automatic detection algorithm for pharyngeal airway on OSA patient's CBCT data using an artificial intelligence system that will provide an easy, errorless and fast method. The second aim is to validate the newly developed artificial intelligence system in comparison to commercially available software for 3D CBCT evaluation.
**Methods and materials:** AI based Convolutional Neural Network based machine learning algorithm (Diagnocat) was created and used for the segmentation of the pharyngeal airways in OSA and non-OSA patients. Radiologists used a semi-automatic software to manually determine the airway and their measurements were compared with the AI. OSA patients were classified as minimal, mild, moderate and severe groups and the mean airway volumes of the groups were compared. Narrowest points of the airway (mm), field of the airway (mm2) and volume of the airway (cc) of both OSA and non-OSA patients were also compared.
**Result:** According to the results of the AI segmentation and manual segmentation, a successful software which can automatically and precisely segment the pharyngeal airway was created. Comparative results showed no statistically significant airway volume (cc) measurement difference between the manual technique and Diagnocat (AI) in all OSA severity subtypes ($p>0.05$). There was no statistically significant difference in narrowest points (mm), airway area ($mm^2$) and total airway volume (cc) measurements between the manual and AI in Non-OSA patients. There were, also, no statistically significant difference in narrowest points (mm), airway area ($mm^2$) and total airway volume (cc) measurements between the manual and AI in OSA patients. The mean value for the narrowest distance was found 6.31 mm with the manual technique and 6.10 mm with AI. The mean value for the airway area was found 1057.59 $mm^2$ with the manual technique and 1013.90 $mm^2$ with AI. The mean value for the total airway volume was found 19.63 cc with the manual technique and 20.25 cc with AI.
**Conclusion:** According to the results of this study an AI segmentation and manual segmentation, a successful software which can automatically and precisely segment the pharyngeal airway was created. Besides, the AI software can able to detect OSA patients and classify precisely.

### Diagnostic accuracy of CT in neck infections: A systematic review and meta-analysis of positive predictive value

*Authors:* *J. Hagelberg[1], B. Pape[2,3], J. Heikkinen[4], J. Nurminen[4], K. Mattila[4], J. Hirvonen[1,4]; [1]University of Tampere, Department of Radiology, Tampere, Finland, [2]Turku University Hospital, Turku Clinical Research Center, Turku, Finland, [3]University of Vaasa, School of Technology and Innovations, Vaasa, Finland, [4]University of Turku, Department of Radiology, Turku, Finland*

**Purpose/Objectives:** Contrast-enhanced computed tomography (CT) is considered the standard imaging modality in neck infections because of good availability and low cost. However, diagnostic accuracy of CT varies substantially between studies, and several diagnostic criteria exist for the diagnosis of an abscess, including low-density core, rim enhancement, bulging, or scalloping. This study reviewed the diagnostic accuracy of contrast-enhanced computed tomography (CT) in differentiating abscesses from cellulitis in patients with neck infections, using surgical findings as reference standard.
**Methods and materials:** Previous studies in the last 30 years were searched from PubMed and Embase. Because of partial verification bias (only positive abscess findings are usually verified surgically), sensitivity and specificity estimates are unreliable, and we focused on positive predictive value (PPV). For all studies, PPV was calculated as the proportion of true positives out of all positives on imaging. To estimate pooled PPV, we used both median with interquartile range (IQR), and a model-based estimate in a meta-analysis. For narrative purposes, we reviewed the utility of common morphological CT criteria for abscesses, such as central hypodensity, large size of collection, bulging, rim enhancement, and presence of air, as well as sensitivity and specificity values reported by the original reports.
**Result:** 23 studies were found reporting 1452 patients, 14 studies in children (771 patients), two in adults (137 patients), and seven including all ages (545 patients). PPV ranged from 0.67 to 0.97 in individual studies, had a median of 0.84 (IQR 0.79–0.87), and model-based pooled estimate of 0.83 (95 % confidence interval 0.80–0.85).

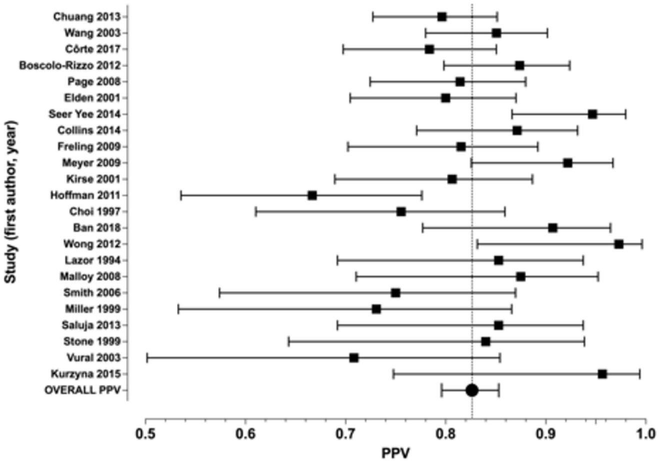

Forest plot of PPV values from individual studies and model-based pooled estimate of PPV. Bars represent 95% confidence intervals.

Six studies reported sensitivity and specificity, and two additional studies reported sensitivity alone.
Sensitivity ranged from 0.68 to 0.99, and specificity from 0.18 to 0.63. Most morphological CT criteria had considerable overlap between abscesses and cellulitis.
**Conclusion:** The pooled estimate of PPV is 0.83 for diagnosing neck abscesses with CT. False positives may be due to limited soft tissue contrast resolution. Overall, none of the morphological criteria seem to be highly accurate for differentiation between abscess and cellulitis.

### Endodontic working length measurements of premolars and molars in high resolution dental MRI: A clinical pilot study for assessment of reliability and accuracy

*Authors: M. Zidan[1], F. S. Schwindling[2], A. Juerchott[1], J. Mente[3], H. Gehrig[3], M. Nittka[4], Z. Hosseini[5], J. M. E. Jende[1], S. Heiland[1], M. Bendszus[1], T. Hilgenfeld[1]; [1]University Hospital Heidelberg, Neuroradiology, Heidelberg, Germany, [2]Heidelberg University Hospital, Prosthodontics, Heidelberg, Germany, [3]Heidelberg University Hospital, Conservative Dentistry, Heidelberg, Germany, [4]Siemens Healthcare GmbH, Magnetic Resonance, Erlangen, Germany, [5]Siemens Healthcare GmbH, Magnetic Resonance, Atlanta, United States*

**Purpose/Objectives:** To prospectively assess the reliability and accuracy of high-resolution, dental MRI (dMRI) for endodontic working length (WL) measurements of premolars and molars under clinical conditions.
**Methods and materials:** Three-Tesla dMRI was performed in 9 subjects who also had undergone cone-beam computed tomography (CBCT) (mean age: 47 ± 13.5 years). A total of 34 root canals from 12 molars (4/8, upper/lower jaw; 22 root canals) and 11 premolars (2/9 upper/lower jaw; 12 root canals) were included. CBCT and dMRI datasets were reconstructed to visualize the root canal in one single slice. Subsequently, two radiologists measured the root canal lengths in both modalities twice in blinded fashion. Reliability and accuracy for both modalities were assessed using intraclass correlation coefficients (ICCs) and Bland-Altman analysis respectively.

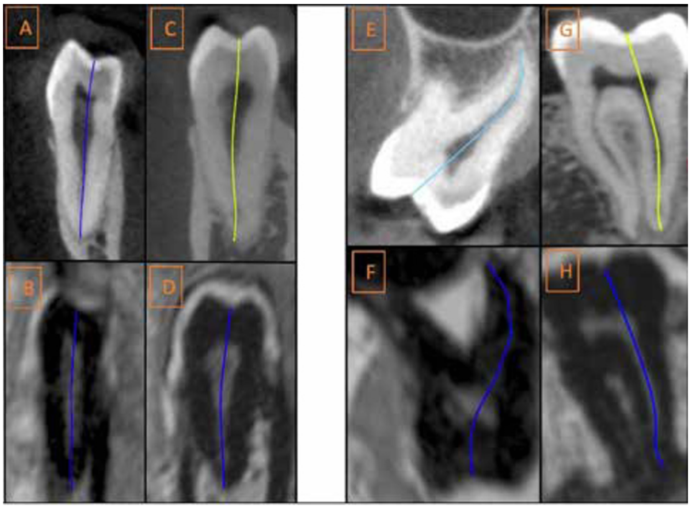

Working length measurements in CBCT and dMRI for premolars (left, A-D) and molars (right, E-H); a first left lower (A-B) and first right lower premolar (C-D); a second left upper molar (E-F) and second left lower molar (G-H)

**Result:** Reliability (intra-rater I/II; inter-rater) of dental MRI measurements was excellent and comparable to CBCT for premolars (0.993/0.900; 0.958 vs. 0.993/0.956; 0.951) and for molars (0.978/0.995; 0.986 vs. 0.992/0.996; 0.989). Bland-Altman analysis revealed a mean underestimation/bias (95 % confidence interval) of dMRI measurements of 0.8 (-1.44/3.05) mm for premolars and 0.4 (-1.55/2.39) mm for molars. In up to 59 % of the cases, the accuracy of dMRI for WL measurements was within the underestimation margin of 0 mm to 2 mm short of the apical foramen AF.
**Conclusion:** *In vivo* demonstration and measurement of WL are feasible using dMRI. The reliability of measurements is high and equivalent to CBCT. Nonetheless, due to lower spatial resolution and longer acquisition time, the accuracy of dMRI is inferior to CBCT, impeding its current use for clinical treatment planning.

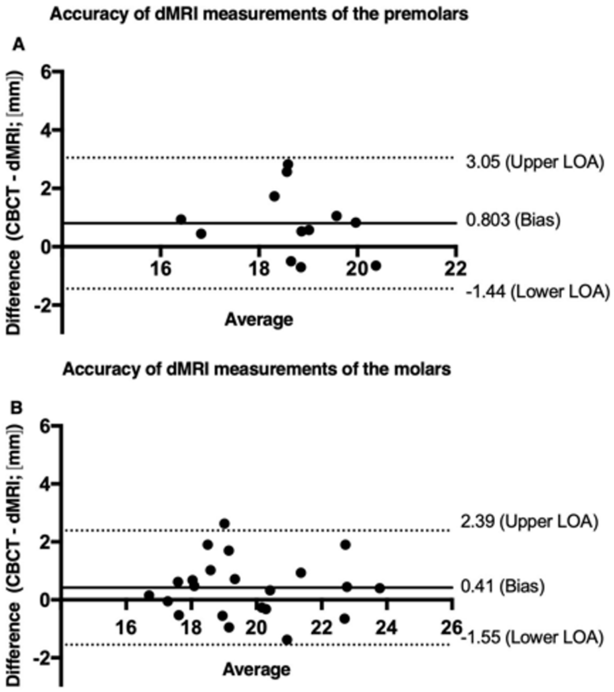

Bland-Altman plots of the mean differences between CBCT and dMRI for working length measurements for premolars (A) and molars (B) illustrating an underestimation of dMRI derived measurements for both premolars and molars.

### Evaluation of dental pathologies and their complications encountered during the routine head and neck CT examination

Author: N. Slavkova; St. Ekaterina University Hospital, Sofia, Bulgaria

**Purpose/Objectives:** To investigate the incidence of dental pathologies detected on routine examination of the head and neck, held because of different reasons, and to show the radiologist's role in preventing further dental complications by making a report that includes detailed dental status.
**Methods and materials:** For this retrospective study we used dynamic volume CT with 320-detector-rows. 77 patients (17-88 years) underwent multiphase, contrast-enhanced CT examination. There were different reasons for performing the examination, the most common for the assessment of benign and malignant tumors of the head and neck (local status, regional tumor spread, staging, and posttreatment changes). Each report included dental status and the detected findings were classified as tooth loss, dental restorations or procedures, and dental pathologies (carious lesions, periapical lesions, periodontal diseases, peri-prosthetic infections, osteomyelitis or osteonecrosis of the adjacent bone, odontogenic sinusitis). The proportion of findings was reported as simple percentiles.
**Result:** Seventy-seven CT scans were included in the study. Dental restorations or procedures were determined in 29 (37.0 %) patients. A total of 22 (28.0 %) patients had normal teeth count and morphology. A total of 35 (45.0 %) patients had at least one tooth loss. A total of 53 (68 %) patients had at least one or more dental pathology. The number of dental carious lesions, periapical lesions, periodontal disease, peri-prosthetic infections, osteomyelitis or osteonecrosis of the adjacent bone, and odontogenic sinusitis were 15 (19.0 %), 15 (19.0 %), 10 (12 %), 7 (10.0 %) and 10 (12.0 %), respectively.
**Conclusion:** Although dental CT is not routinely performed at hospital imaging centers, dental and periodontal disease can be recognized on standard high-resolution CT of the head and neck. Recognition of dental and periodontal disease has the potential to affect management and preclude further complications. So radiologists can play an important role in the early diagnosis of dental diseases like caries, or periodontal disease and prevent further complications such as chronic periodontitis and abscesses, nonhealing fractures, osteomyelitis, and oroantral fistulas.

### Guidelines for Magnetic Resonance Imaging in pediatric head and neck pathologies: A multicentre international consensus paper

Authors: F. D'Arco[1], L. Mertiri[2], B. De Foer[3], D. Farina[4], A. Siddiqui[5], S. Bisdas[6], S. Van Cauter[7], K. Popovic[8], M. Severino[9], K. Mankad[1], H. Brisse[10], P. de Graaf[11], M. Argyropoulou[12], A. Juliano[13], S. Connor[5]; [1]Great Ormond Street Hospital, Radiology, LONDON, United Kingdom, [2]La Sapienza University, Rome, Italy, [3]GZA Hospitals, Radiology, Antwerp, Belgium, [4]Brescia University Hospital "Spedali Civili", Radiology, Brescia, Italy, [5]King's Hospital, Radiology, London, United Kingdom, [6]Queen's square hospital, Radiology, London, United Kingdom, [7]Ziekenhuis Oost-Limburg, Medical Imaging, Genk, Belgium, [8]University Medical Center, Radiology, Ljubljana, Slovenia, [9]Gaslini Hospital, Neuroradiology, Genova, Italy, [10]Institut Curie, Paris, France, [11]Cancer Center Amsterdam, Radiology, Amsterdam, Netherlands, [12]University of Ioannina, Radiology, Ioannina, Greece, [13]Mass Ear and Ear, Boston, United States

**Purpose/Objectives:** The use of standardized imaging protocols is paramount in order to facilitate comparable, reproducible images and, consequently, to optimize patient care. Standardized MR protocols are lacking when studying head and neck pathologies in the pediatric population. We propose an international, multicentre consensus paper focused on providing the best combination of acquisition time/technical requirements and image quality. Distinct protocols for different regions of the head and neck and, in some cases, for specific pathologies or clinical indications are recommended. This white paper is endorsed by several international scientific societies and is the result of discussion, in consensus, among experts in pediatric head and neck imaging.
**Methods and materials:** We first assessed the variability of head and neck protocols across institutions in Europe and North America, and then provided a consensus based on the opinion of the members of different European radiology societies and specific committees.
A first paper version was drafted by the co-first authors (FD'A and LM) and the last author (SB) after agreeing on the paper's main paragraphs and a search of the relevant literature. The draft was subsequently circulated among the co-authors and collaborators of the COMPS group (which includes pediatric radiologists, neuroradiologists, radiologists with special interest in head and neck imaging, MR physicists, oncologists, surgeons and radiographers) and representatives of the endorsing scientific societies. After all comments were received, they were either integrated in the revised manuscript or, when discordant opinions were present, they were resolved by consensus. The final manuscript was again shared with the group.
**Result:** We propose specific MR protocols for each head and neck region and, in some cases, for specific diseases, with a range of technical parameters. Optional sequences are also added.
**Conclusion:** MR imaging in pediatric head and neck pathology is very complex, it depends on the region of interest and poses several challenges. We propose standardized MR protocols to help the readers in approaching pediatric head and neck pathologies and to help minimize the variability and maximize diagnostic efficiency. We discussed the added value of CT in specific areas or diseases, basics and optional/alternative MR sequences depending on the clinical problem/region of interest. This consensus paper has been endorsed by many international scientific societies. The members of the COMPS group recognize that this is intended as a work-in-progress, and further improvement and updates of the present consensus statement will be necessary overtime.

### High-frequency intraoral ultrasound for the preoperative assessment of the depth of invasion (DOI) for early tongue squamous cell carcinoma

*Authors:* *S. Caprioli[1], A. Casaleggio[2], A. Tagliafico[3, 4], F. Borda[4], M. Fiannacca[4], C. Conforti[2], G. Cittadini[5]; [1]University of Genova, DIMI, Genova, Italy, [2]IRCCS Ospedale Policlinico San Martino, Radiologia Interventistica, Genova, Italy, [3]IRCCS Ospedale Policlinico San Martino, Radiologia, Genova, Italy, [4]University of Genova, DISSAL, Genova, Italy, [5]IRCCS ospedale policlinico San Martino, Radiologia Generale, Genova, Italy*

**Purpose/Objectives:** In 2017, the 8th edition of the TNM classification officially introduced the "depth of invasion" (DOI) as parameter for the local staging of oral cancer. Imaging has a crucial role for preoperatively assess DOI, thus for programming the surgical approach. Recently, a growing interest has been developing for the use of intraoral ultrasound (IOUS) in the local staging of oral squamous cell carcinoma (SCC) as an alternative to magnetic resonance imaging (MRI), especially for early-stage SCC of the oral tongue. The aim of this work is to demonstrate the accuracy of IOUS in the assessment of DOI in early oral SCC (CIS, pT1 and pT2).
**Methods and materials:** 41 patients with tongue SCC (CIS-T2) underwent preoperative high frequency IOUS. IOUS was performed using a small-size, high-frequency hockey-stick linear probe (22-8 MHz). Ultrasonographic DOI (usDOI) was compared to pathological DOI (pDOI) as reference standard. For the patients who underwent preoperative MRI, usDOI, pDOI and magnetic resonance DOI (mriDOI) were compared.

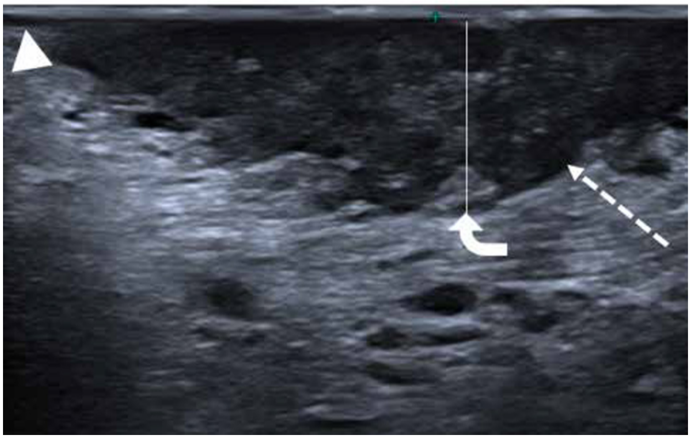

High frequency ultrasound of a squamous cell carcinoma of the lateral surface of the oral tongue (dashed arrow). Ultrasonographic DOI is measured perpendicularly from the mucosal surface to the deepest point of infiltration (curved arrow), using the closest normal mucosa as reference line (arrowhead)

**Result:** High correlation was found between pDOI and usDOI, pDOI and mriDOI and usDOI and mriDOI (ρ=0,84, P<0,0001, ρ=0,79, P<0,0001 and ρ=0,91, P<0,0001 respectively). Bland-Altmann plot showed a high agreement between usDOI and pDOI, even though a mean systematic error was found between usDOI and pDOI (0,7 mm), between mriDOI and pDOI (1,6 mm) and between usDOI and mriDOI (-0,7 mm). IOUS was accurate in determining the T-stage (P<0,0001).

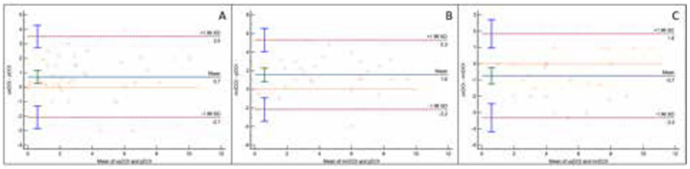

Blant Altman plots comparing usDOI and pDOI (A), mriDOI and pDOI (B) and usDOI and mriDOI (C).

**Conclusion:** IOUS is accurate in the preoperative assessment of pDOI and T-stage and could be proposed as an alternative to MRI in the preoperative staging of early tongue SCC.

### Is gadolinium needed for MRI diagnosis of tonsillar infections?

*Authors:* *J. Hirvonen[1, 2], V. Jussila[2], J. Nurminen[2], M. Nyman[2], J. Heikkinen[2], K. Mattila[2]; [1]University of Tampere, Department of Radiology, Tampere, Finland, [2]University of Turku, Department of Radiology, Turku, Finland*

**Purpose/Objectives:** MRI has excellent diagnostic accuracy in tonsillar infections, but scan protocols are long and intravenous gadolinium-based contrast agent is often required to delineate abscesses. A shorter (5 min) scan protocol including only T2-weighted and diffusion-weighted (DWI) sequences would be beneficial for the emergency setting. We assessed the diagnostic accuracy of this short protocol in patients with tonsillar infections.
**Methods and materials:** Three board certified radiologists retrospectively analyzed MRI images from 52 patients with acute tonsillar infections (13 tonsillitis with no abscess, 19 peritonsillar abscesses, and 20 deeply extending abscesses) for absence or presence of an abscess, and for deep extension into parapharyngeal or retropharyngeal spaces. Data were analyzed twice, in two separate sessions at least two months apart: first, with only axial T2-weighted Dixon and DWI images; and second, also including axial, coronal, and sagittal T1-weighted post-contrast Dixon images. Readers were blind to clinical and surgical data, and cases were re-randomized between reading sessions to avoid carry-over effects from previous reading. Diagnostic accuracy was calculated using surgical findings as reference, and interobserver agreement using Fleiss' Kappa.
**Result:** Overall diagnostic accuracy for abscesses across all readers was good to excellent using the short protocol: sensitivity 0.93, specificity 0.85, positive predictive value (PPV) 0.95, negative predictive value (NPV) 0.80, accuracy 0.91. Adding T1-weighted post-contrast images slightly improved these numbers: sensitivity 0.98, specificity 0.85, PPV 0.95, NPV 0.94. Regarding detection of deep extension, accuracy improved marginally from 0.85 to 0.87, whereas specificity improved from 0.92 to 0.97, by adding contrast-enhanced images. Interobserver Kappa improved from 0.70 to 0.82 for abscesses and from 0.50 to 0.66 for deep extension by adding T1-weighted post-contrast images.

| | No contrast | | With contrast | |
|---|---|---|---|---|
| | Abscess | Deep extension | Abscess | Deep extension |
| Sensitivity | 0.93 | 0.73 | 0.98 | 0.70 |
| Specificity | 0.85 | 0.92 | 0.85 | 0.97 |
| Positive predictive value | 0.95 | 0.85 | 0.95 | 0.92 |
| Negative predictive value | 0.80 | 0.85 | 0.94 | 0.84 |
| Accuracy | 0.91 | 0.85 | 0.95 | 0.87 |

Diagnostic accuracy for abscesses and deep extension with and without contrast agent.

**Conclusion:** Even a short 5 min MRI protocol including only T2-weighted and DWI sequences with no gadolinium-based contrast agent has good to excellent diagnostic accuracy for pharyngotonsillar abscesses, outperforming previously published estimates for contrast-enhanced CT. Adding T1-weighted post-contrast images reduced false negatives for abscesses and false positives for deep extension, and improved radiologist agreement. Thus, the choice to give a gadolinium-based contrast agent for emergency MRI for suspected pharyngotonsillar infections is a trade-off between significantly shorter scan times and slightly improved accuracy.

### Long Term Follow-up of Incidental Thyroid Nodules on Computed Tomography Imaging

*Authors: E. Lyuman[1], C. McArthur[2]; [1]University of Glasgow, School of Medicine, Glasgow, United Kingdom, [2]Glasgow Royal Infirmary, Radiology, Glasgow, United Kingdom*

**Purpose/Objectives:** Reported prevalence of incidental thyroid nodules (ITNs) on CT is 5-25 %. Nodule malignancy rate is 5-15 % but prognosis in differentiated thyroid cancer is excellent. Thus, ITNs pose a common management dilemma. The aims of the study are to determine prevalence of ITNs ≥1 cm on CT images based on data from west of Scotland in 2009, evaluate reporting practices and examine short, medium and long-term outcomes including rate of clinically evident thyroid cancer at 13-year follow-up, interim nodule growth and clinical outcomes in nodules that were further investigated.

**Methods and materials:** 1499 consecutive CT scans in adults that included the thyroid, performed during a 19-day period in January 2009, in Greater Glasgow & Clyde National Health Service Board were evaluated. Clinical data up to January 2022 was analyzed in 150 included patients with at least one ITN ≥1 cm. Statistical analysis was performed with Minitab version 1 9.2020.2.0.

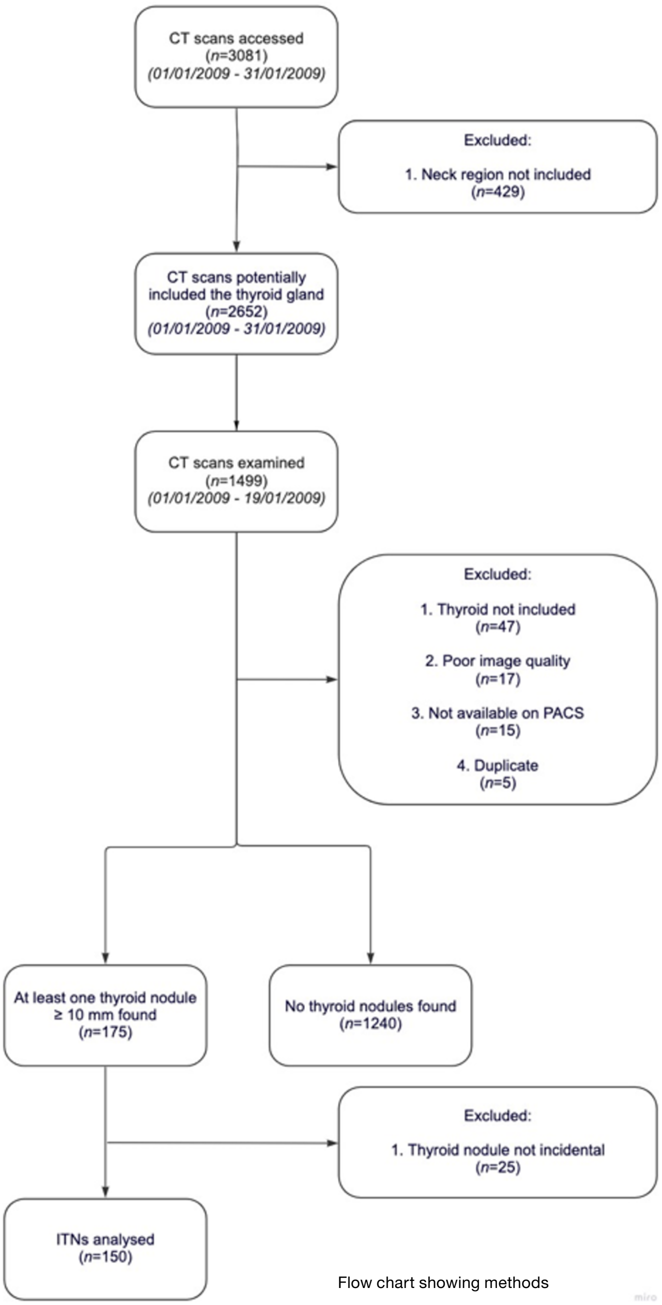

Flow chart showing methods

**Result:** 69 % of (n=104) patients were female and 31 % (n=46) were male. Mean age at the time of CT was 70 (±12.06) years. ITN prevalence in the study sample was 10.7 %.

Subsequently, 11.3 % had thyroid US, 5.3 % FNA and 2 % diagnostic hemithyroidectomy with no thyroid malignancy found.

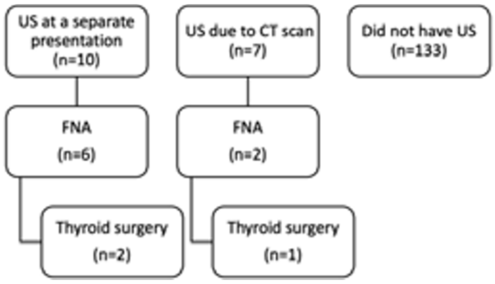

Figure showing further investigations and procedures performed in the follow-up period for the ITNs detected in the study

80 % of the patients (n=120) were deceased by the study endpoint, all from other causes.

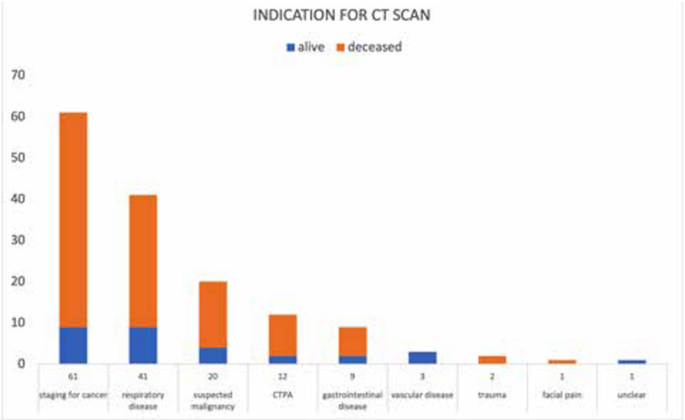

Bar chart showing indication for CT scan stratified by health status (alive/deceased) by study endpoint

30 % (n=45) of the ITNs were reported in radiology reports.

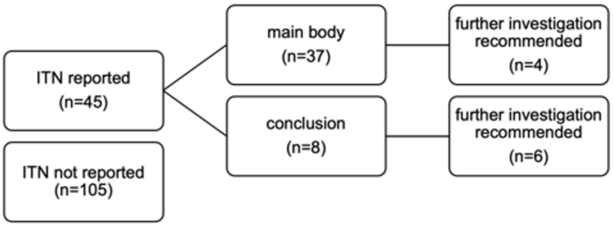

Bar chart showing indication for CT scan stratified by health status (alive/deceased) by study endpoint

56 % (n=84) of patients had further cross-sectional imaging that covered the thyroid in the follow-up period. In 70 % (n=59) of these cases, the nodule size did not change. In 14 % (n=12), the nodule had increased in size. In 9.5 % (n=8), the nodule had disappeared. In 6 % (n=5), the nodule had decreased in size.

**Conclusion:** In our study, none of the 150 ITN cases presented with or were shown to have thyroid malignancy in the 13-year follow-up period. All cases that were further investigated were confirmed to be benign. 80 % of patients were deceased by the study endpoint from non-thyroid causes. This study would support a pragmatic approach and that ITNs detected on CT scan in daily practice do not require further investigation unless invasive features are present or there is significant clinical concern for thyroid malignancy.

### MR Imaging Characteristics of Uveal Melanoma with Histopathological Validation

*Authors: T. Ferreira[1], M. G. Jaarsma-Coes[2], M. Marinkovic[3], B. Verbist[1], R. Verdijk[4], M. Jager[3], G. Luyten[3], J.-W. Beenakker[2]; [1]LUMC, Radiology, Leiden, Netherlands, [2]LUMC, Radiology, Ophthalmology, Leiden, Netherlands, [3]LUMC, Ophthalmology, Leiden, Netherlands, [4]LUMC, Erasmus MC University Medical Center, Pathology, Leiden, Rotterdam, Netherlands*

**Purpose/Objectives:** To evaluate the MRI characteristics of UM, to compare them with fundoscopy and Ultrasound (US) and to validate them with histopathology.
**Methods and materials:** MR-images from 42 UM were compared with US and fundoscopy, and on 14 enucleated cases with histopathology.
**Result:** A significant relationship between the signal intensity on T1 and pigmentation on histopathology was found (p=0.024). Mean Apparent Diffusion Coefficient of the UM was 1.16 ± 0.26 x $10^{-3}$ $mm^2/s$. Two-thirds of the UM had a wash-out and the remaining a plateau perfusion time-intensity curve. MRI was limited in evaluating the basal diameter of flat tumors. US tends to show larger tumor prominence (0.5 mm larger, p=0.008) and largest basal diameter (1.4 mm larger, p<0.001). MRI was good in diagnosing ciliary body involvement, extrascleral extension and optic nerve invasion, but limited on identifying scleral invasion. UM with loops tended to have a shorter Arrival time, a shorter Time to peak and higher Peak intensities.
**Conclusion:** Knowing the MRI characteristics of UM is important in order to confirm the diagnosis, to differentiate UM from other intra-ocular lesions and because it has implications for treatment planning. MRI is a good technique to evaluate UM, being only limited in case of flat tumors or on identifying scleral invasion. Perfusion has a promising value in recognizing UM with a poor prognosis.

### MR Imaging Phenotype of *MYCN*-amplified *RB1* wild-type Retinoblastoma

*Authors: R. Jansen[1], C. de Bloeme[1], H. Brisse[2], S. Göricke[3], P. Maeder[4], S. Sirin[3], L. Cardoen[5], P. Galluzzi[6], S. van Elst[1], A. Ramasubramanian[7], A. Skalet[8], A. Miller[8], O. Uner[9], B. Hubbard[9], C. Hinz[10], C. Boldt[10], R. Brennan[11], S. Sen[12], J. Dorsman[1], P. van der Valk[1], R. Boellaard[1], A. Moll[1], J. Castelijns[1], M. de Jong[1], P. de Graaf[1], European Retinoblastoma Imaging Collaboration; [1]Amsterdam UMC, location VUmc, Amsterdam, Netherlands, [2]Insitut Curie, Radiology, Paris, France, [3]Essen University Hospital, Radiology, Essen, Germany, [4]Centre Hospitalier Universitaire Vaudois, Radiology, Lausanne, Switzerland, [5]Institut Curie, Radiology, Paris, France, [6]Azienda Ospedaliera Universitaria Senese, Radiology, Siena, Italy, [7]Phoenix Children's Hospital, Phoenix, United States, [8]Oregon Health & Science University Hospital, Portland, United States, [9]Emory University Hospital, Atlanta, United States, [10]University of Iowa Hospitals & Clinics, Iowa City, United States, [11]St. Jude Children's Research Hospital, Memphis, United States, [12]Tata Medical Center, Kolkata, India*

**Purpose/Objectives:** The purpose of this study was to define the MR imaging phenotype of $MYCN^{ampl}RB1^{+/+}$ retinoblastoma.
**Methods and materials:** In this retrospective multicenter case-control study MR images of $MYCN^{ampl}RB1^{+/+}$ retinoblastoma cases and *RB1*-driven controls were collected in a ratio of 1:4. Inclusion criteria were (1) histopathologically confirmed unilateral retinoblastoma with genetic testing for *RB1*-mutation and *MYCN*-amplification and MR imaging performed (after 1995). Associations between radiologist-scored imaging features and diagnosis were assessed with exact tests corrected for multiple hypothesis testing. Additionally, manual delineations were performed to enable extraction of radiomic features, which were incorporated in a random forest model.
**Result:** From 10 retinoblastoma referral centers, 110 retinoblastoma patients were included: 22 $MYCN^{ampl}RB1^{+/+}$ cases and 88 controls (mean age 12 months +/- 11 [standard deviation]; 51 [46 %] female). $MYCN^{ampl}RB1^{+/+}$+/+ retinoblastomas are more anteriorly located, are often plaque or pleomorphic shaped, have irregular margins, and show extensive retina folding with vitreous enclosure, peritumoral blood, and strong anterior eye segment enhancement (all corrected $P < 0.05$). A radiomics-based analysis was able to distinguish the $MYCN^{ampl}RB1^{+/+}$ subtype with an area under the curve of 0.70.
**Conclusion:** MR imaging features can be adopted to recognize the more aggressive $MYCN^{ampl}RB1^{+/+}$ retinoblastoma which may improve the selection for tailored treatment in the future.

### MRI features of spontaneous lateral temporal bone cephaloceles

*Authors: R. Srinivasan[1], R. Obholzer[2], S. Connor[1]; [1]Guy's & St. Thomas' Hospitals NHS Foundation Trust, Clinical imaging and Medical Physics, London, United Kingdom, [2]Guy's & St. Thomas' Hospital NHS Foundation Trust, Otolaryngology, London, United Kingdom*

**Purpose/Objectives:** To determine the clinical presentation, location and MRI features of spontaneous lateral temporal bone cephaloceles.
Spontaneous cephaloceles of the lateral temporal bone are an important and emerging clinical phenomenon. Diagnosis is often challenging due to the non-specific symptoms including hearing loss and middle ear fullness. Prompt and accurate diagnosis is essential as patients are at risk of meningitis. Imaging can have a crucial role in its diagnosis.
**Methods and materials:** A retrospective cohort study analysed patients with evidence of lateral temporal bone cephaloceles on imaging as defined by CSF or cerebrum traversing the tegmen tympani or mastoideum. Cases were identified from radiology and surgical databases between 01/01/2006 to 09/02/2022. Non–spontaneous cephaloceles and patients without 3D T2W imaging were excluded. Data collection included demographic information, presenting signs and symptoms, surgical history. Two head and neck radiologists analysed MRI pre-defined features.
**Result:** Thirty-one patients were included (M=14, F=17; mean age 56.9, age range 31-88 years). Five patients (16.1 %) had bilateral defects. The total cohort included 36 lateral temporal bone cephaloceles. Twenty-two patients (61.1 %) underwent surgical repair. Hearing loss (67.7 %) was the most common symptom and 4 patients (12.9%) had meningitis.
High T2W mastoid signal was found in 32/36 (88.9 %) of all cases and 21/22 (95.4 %) surgically confirmed cases. MRI Imaging features of intracranial hypertension were seen in 9/36 (32.3 %) of all cases and 6/22 (27.2 %) surgically confirmed cases. The tegmen tympani was the most common site of defect found in 17/36 (47.2 %) of all cases and 11/22 (50%) surgically confirmed cases. A high T2W CSF cleft, in continuity with an overlying sulcus, was identified in 22/36 (61.1 %) cases overall and in 13/22 (59.1 %) surgically confirmed cases. High T2W signal, at the margin or pointing towards the defect, was detectable in the 13/14 (92.8 %) of the remaining total cases and in 8/9 (88.9 %) of the remaining surgically confirmed cases.
**Conclusion:** The principle clinical and MRI features of spontaneous lateral temporal bone cephaloceles are described with a view to aiding their identification. A high T2W CSF cleft, either at the margin or in continuity with an overlying sulcus traversing the defect, is a potentially useful diagnostic feature.

### MRI in the evaluation of the Eustachian tube cartilage in chronic otitis media

*Authors: U. Abdullaeva, M. Khodjibekov; Hospital of the Tashkent Medical Academy, Radiology, Tashkent, Uzbekistan*

**Purpose/Objectives:** In this paper, dimensions (length, thickness and volume) and anatomical changes (margins, structure) of the Eustachian tube cartilage (ETC) in patients with chronic otitis media (COM) using MRI are studied. The dimensions and anatomical parameters of ETC measured according to MRI data in patients with COM were compared with the control group and revealed their significant changes ($p<0.001$).
**Methods and materials:** The study was performed in 1.5 T MRI in 56 Eustachian tubes (ET) in patients with COM obtaining axial and oblique parasagittal planes of ETC in T2FS, PDW and T1W images.

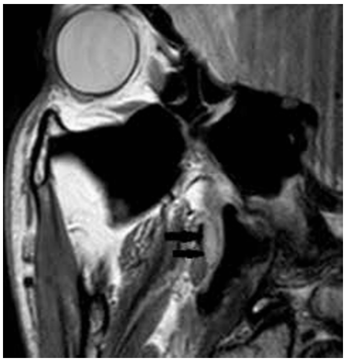

Slightly hyperintense signal of the ET cartilage (arrows) is shown in oblique parasagittal PDW image in the in a healthy person

Patients were aged 13 to 60 years. As a control group, data from MRI studies of 36 ET without middle ear pathology were used (individuals aged from 20 to 42 years).
The anatomical parameters of the ET cartilage, such as length, thickness, and volume, were evaluated. ET cartilage margins and structure were assessed in T2FS and PDW images.
**Result:** Significant differences in the frequency of ETC anatomical parameters ($p<0.001$) were established in patients with COM, such as margins irregularity (60.7±6.53 %), structural heterogeneity (75.0±5.79 %), compared with the control group (19.2±7.73 % and 11.5±6.27 % respectively).
The average values of the length, thickness and volume of the CT cartilage in patients with COM were 1.37±0.03 cm, 0.39±0.01 cm and 0.41±0.02 $cm^3$, in the control group 1.63±0.04 cm, 0.49±0.02 cm and 0.7±0.05 $cm^3$, respectively. When comparing the data of both groups, a significant decrease in ETC parameters was found in patients with chronic otitis media ($p<0.001$).

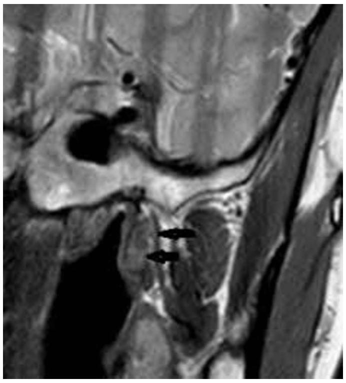

Diffuse heterogeneity of the ET cartilage (arrows) is shown in oblique parasagittal PDW image in a patient with COM

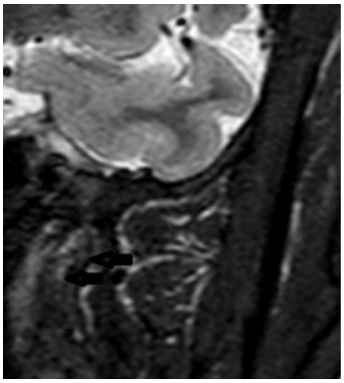

Diffuse heterogeneity, margins irregularity and reducing the size of the ET cartilage (arrows) are shown in oblique parasagittal T2FS image in a patient with COM

**Conclusion:** To sum up, in patients with COM, changes in the parameters of the ETC appear to be distinguishable from the healthy people, which indicates a significantly smaller size of the cartilage in patients. The high frequency of occurrence of the anatomical changes of the ETC indicates degenerative changes in the cartilage, which should also play an important role in the functioning of the tube. The revealed morphological changes of the ETC may prevent the adequate functioning of the ET, aggravating the course of the inflammatory process in the middle ear.

### MRI of odontogenic neck infections: diagnostic accuracy and reliability

*Authors: J. Heikkinen[1], V. Jokihaka[1], J. Nurminen[1], V. Jussila[1], J. Velhonoja[2], H. Irjala[2], T. Soukka[3], T. Happonen[1], J. Järnstedt[4], M. Nyman[1], K. Mattila[1], J. Hirvonen[1, 4]; [1]University of Turku and Turku University Hospital, Department of Radiology, Turku, Finland, [2]University of Turku and Turku University Hospital, Department of Otorhinolaryngology-Head and Neck Surgery, Turku, Finland, [3]University of Turku and Turku University Hospital, Department of Oral and Maxillofacial Surgery, Turku, Finland, [4]Tampere University and Tampere University Hospital, Department of Radiology, Tampere, Finland*

**Purpose/Objectives:** To determine the diagnostic accuracy of emergency magnetic resonance imaging (MRI) in odontogenic neck infections, clinical and surgical significance of MRI findings, and whether MRI can identify the acute tooth responsible for the infection.
**Methods and materials:** A retrospective cohort study reviewed 106 emergency neck MRI scans of patients with neck infections of odontogenic origin. We studied whether MRI can accurately identify the abscess relative to surgical findings, and correlated various MRI edema patterns and abscess diameters with clinical results and outcomes, such as the surgical approach (intraoral vs. extraoral). Ability of MRI findings to predict the causative tooth was assessed in a blinded multi-reader setting.
**Result:** Of the 106 patients with odontogenic infections, 77 (73 %) had one or more abscesses. Imaging showed sensitivity, specificity, and accuracy of 0.95, 0.84, and 0.92, respectively, for MRI diagnosis of an odontogenic abscess. MRI showed bone marrow edema in the majority of patients, and multi-reader assessment showed good reliability. MRI was also able to predict the causative tooth accurately. Among the imaging findings, mediastinal edema was the strongest predictor of extraoral surgery.

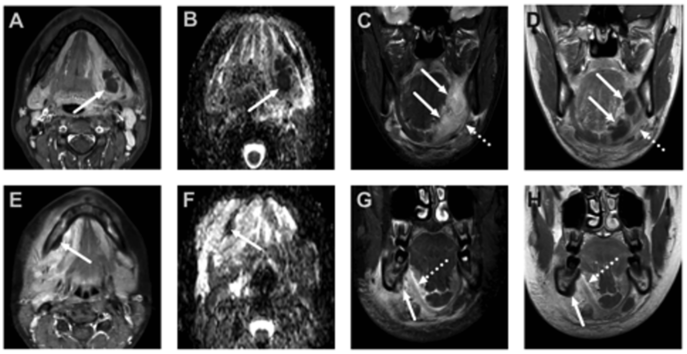

Sublingual (upper row) and submandibular (lower row) space abscess and relation to the mylohyoid muscle (dotted arrows)

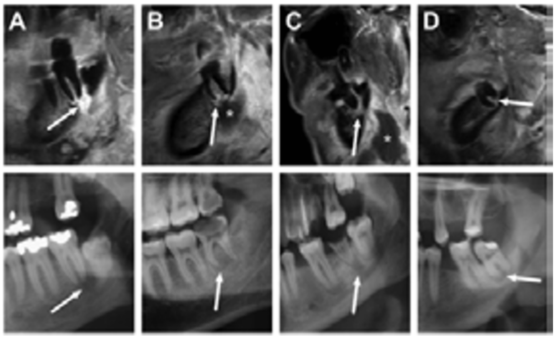

Four patients with odontogenic deep neck infection, periapical infection on T1 fat-suppressed sagittal images with Gadolinium (upper row), and corresponding DPR findings (lower row).

| Radiologists agreeing | Interobserver | MRI vs. Reference standard | MRI vs. DPR |
|---|---|---|---|
| at least 1 of 3 | N.A. | 96 % | 91 % |
| at least 2 of 3 | 96 % | 83 % | 77 % |
| 3 of 3 | 61 % | 57 % | 55 % |

Degree of agreement in radiologists' assessments of MRI focus tooth compared to reference standard and dental panoramic radiography

**Conclusion:** Emergency neck MRI can accurately detect odontogenic abscesses, and reliably point to the causative tooth. These results can increase the utility and reliance on emergency MRI in clinical decision-making.

### Predicting Response to Neoadjuvant Immunotherapy in Patients with Locally Advanced Head and Neck Carcinoma using Diffusion-Weighted Imaging Radiomics

*Authors: H. J. van der Hulst[1, 2], J. L. Vos[3, 4, 5], R. Tissier[6], L. A. Smit[7], R. M. Martens[8], R. G. H. Beets-Tan[1, 2, 9], M. W. M. van der Brekel[5, 10], C. Zuur[5, 10, 11, 12], J. A. Castelijns[1]; [1]The Netherlands Cancer Institute, Radiology, Amsterdam, Netherlands, [2]Universiteit of Maastricht, GROW School for Oncology and Developmental Biology, Maastricht, Netherlands, [3]Memorial Sloan Kettering Cancer Center, Immunogenomics and Precision Oncology Platform, New York, United States, [4]Memorial Sloan Kettering Cancer Center, Human Oncology and Pathology Program, New York, United States, [5]The Netherlands Cancer Institute, Department of Head and Neck Surgery, Amsterdam, Netherlands, [6]The Netherlands Cancer Institute, Biostatistics Unit, Amsterdam, Netherlands, [7]The Netherlands Cancer Institute, Pathology, Amsterdam, Netherlands, [8]Amsterdam University Medical Center (AUMC), Radiology and Nuclear Medicine, Amsterdam, Netherlands, [9]University of Southern Denmark, Department of Regional Health Research, Odense, Denmark, [10]Amsterdam University Medical Center (AUMC), Department of Oral and Maxillofacial Surgery, Amsterdam, Netherlands, [11]Leiden University Medical Center,, Department of Otorhinolaryngology Head Neck Surgery, Leiden, Netherlands, [12]The Netherlands Cancer Institute, Division of Tumor Biology and Immunology, Amsterdam, Netherlands*

**Purpose/Objectives:** Neo-adjuvant immunotherapy treatments have shown to be feasible and promising and may improve the survival rates in head and neck squamous cell carcinoma (HNSCC) patients. Delaying surgery in this population, however, requires an accurate and preferably minimally invasive method to reliably establish ICB response. Evaluation of medical imaging according to the current response evaluation criteria in solid tumors (RECIST v1.1) unfortunately underestimates pathological response after neoadjuvant ICB in HNSCC. Functional magnetic resonance (MR) technique of diffusion-weighted imaging (DWI) has proven to be useful for detecting residual or recurrent tumor after chemoradiation. Hence, this study aimed to explore the ability of MR diffusion-based imaging parameters, extracted from MR-imaging acquired at baseline and upon treatment, to predict or timely detect major pathological responses to neoadjuvant ICB in patients with resectable HNSCC.
**Methods and materials:** This retrospective study uses the data from 32 patients with stage II–IV, resectable HNSCC treated within the phase Ib/IIa IMCISON trial (NCT03003637). MR-imaging was acquired at baseline, before ICB (week 0) and after ICB treatment (week 4-5). Treatment consisted of 2 infusions of nivolumab (anti-programmed cell death protein 1) (n=6) or nivolumab with ipilimumab (anti-cytotoxic T-lymphocyte-associated protein 4) ICB (n=26). The entire primary tumor was delineated on the apparent diffusion coefficient (ADC)-map for both time points. 32 features were derived from delineation using PyRadiomics and correlated to the tumor regression percentage based on the surgical specimen (obtained in week 5-6).
**Result:** 2 patients had no DWI available, 6 patients were excluded due to large artefacts or poor DWI quality at the tumor location on pre and post-treatment imaging. Leaving 24 out of the 32 patients to analyse.
Smaller baseline tumor diameter (P=0.01–0.04) and higher sphericity (P=0.03) were significantly predictive of having a good pathological response to ICB. Post-treatment tumor diameter (P=0.04) and skewness (P=0.04) were correlated to the tumor regression percentage upon ICB. The change in skewness between MRI timepoints was likewise negatively correlated with the outcome (P=0.02).
**Conclusion:** The analysis of DWI features identified a significant association between tumor diameter and baseline sphericity and pathological response after neoadjuvant nivolumab and ipilimumab in HNSCC. In addition, ADC skewness may play an important role in pathological response evaluation, either as stand-alone post treatment value or calculated as difference between baseline and post-treatment imaging.

### Radiologic pathologic correlation of depth of invasion (DOI) assessed on Computed Tomography in oral cancer.

*Author: Z. Hussain; Aga Khan University hospital, Karachi, Pakistan*

**Purpose/Objectives:** The eighth edition of the Cancer Staging Manual of the American Joint Committee on Cancer tumor, node, metastases classification has realized the prognostic implication of depth of invasion and has added it to the T staging or oral cancers. although this is ascertained by surgical specimen not all tumors undergo surgery and DOI needs to be determined on imaging. Hence the objective of this study was to assess the correlation of CT based depth of invasion in oral cancers with histopathology as reference standard.
**Methods and materials:** Seventy patients were reviewed from the radiology information systems of the department over a 3-year period till 2018. DOI was evaluated on axial and coronal CT post contrast enhanced images. Patients who had preoperative staging CT scan at the facility as well as histopathology of the surgical specimen were included. Radiological depth of invasion was evaluated from the picture archiving system of the radiology department. Depth of invasion assessed on histopathology was obtained from patient chart review. Data was analyzed on SPSS version 21. Mean for age and depth of invasion on CT and histopathology was estimated whereas frequencies were evaluated for gender. Pearson correlation coefficient was estimated for depth of invasion as calculated on CT and histopathology. P value of <0.05 was considered significant.
**Result:** After filtering patient through the study criteria, 53 patients were recruited in the study. There were 34 (64.2 %) males and 16 (30.2 %) females with age ranging between 31 to 91 years with mean age of 56.58 years with std. dev of 13.4 years. there was a strong correlation between the DOI evaluated on CT scan and histopathology with r=0.853 at a p value of <0.001
**Conclusion:** CT based estimation of depth of invasion is a reliable parameter for staging of oral cancers and should be incorporated in the radiological staging of oral cancers.

### Use of artificial intelligence in management of head-neck tumours: a multidisciplinary survey.

*Authors: C. Giannitto[1], S. Lusi[2], G. Carnicelli[2], G. Mercante[3], G. Spriano[3], M. Francone[2], L. S. Politi[4], L. Balzarini[2]; [1]Humanitas Research Hospital, Head and Neck Imaging, Rozzano, Italy, [2]Humanitas Research Hospital, Radiology, Rozzano, Italy, [3]Humanitas Research Hospital, Otolaryngology Surgery, Rozzano, Italy, [4]Humanitas Research Hospital, Neuroradiology, Rozzano, Italy*

**Purpose/Objectives:** The purpose of this study was to conduct a survey among the specialists involved in the H&N tumour board (radiologists, ENT surgeons, oncologists, radiotherapists) to evaluate the perception, fields of application and interest in AI.
**Methods and materials:** We have developed an online questionnaire consisting of 12 questions, which has been distributed since March 2021 to February 2022 through the European Society of Head and Neck Radiology (ESHNR), the head-neck section of the Italian Medical Radiology Society (SIRM), the Italian Society of ENT and Head and Neck Surgery (SIOeChCF) and social media. The areas investigated concerned personal information, academic roles, the use of AI, perceived relevance and applicative sectors.
**Result:** 102 participants with different levels of experience responded: professors (8.8 %), assistant doctors (45.2 %), residents (31.3 %), doctoral students (14.7 %). 32.3 % of the participants were aged between 30 and 49; 28.6 % between 40 and 49; 16.2 % between 50 and 59; 12.5 % under 30; and 10.37 % over 60 years. Radiologists accounted for the largest share (53.9 %), followed by ENT (36.2 %), oncologists (3.9%), radiotherapists (3.9 %) and bioinformaticians (1.9 %).
The most relevant AI applications were imaging data quantification (70.4 %), diagnosis computer-assisted (46.5 %) and outcome predictions (32.4 %). 33.3 % of the participants considered the use of AI as very important; 50.8% important; 4.5 % essential; 8.3 % not fundamental; and 3 % irrelevant. Participants showed interest in future applications in precision diagnosis and preoperative planning (72 %), together with the choice of more adequate treatment (58.1 %) and time-saving in patient management (52.9 %).
**Conclusion:** The results of this survey demonstrate a relevant interest in AI in the multidisciplinary management of H&N cancers, in particular for precision diagnosis, surgical planning and personalized treatment.

### Volume changes on MR Imaging of retinoblastoma after intra-arterial chemotherapy treatment

*Authors: C. de Bloeme[1, 2], R. Jansen[1, 2], M. de Jong[1, 2], S. van Elst[1, 2], L. Cardoen[2, 3], S. Sirin[2, 4], P. Galluzzi[2, 5], S. Göricke[2, 4], H. Brisse[2, 3], P. Maeder[2, 6], A. Moll[7], P. de Graaf[1, 2]; [1]Amsterdam UMC, Radiology, Amsterdam, Netherlands, [2]European Retinoblastoma Imaging Collaboration (ERIC), Amsterdam, Netherlands, [3]Institut Curie, Paris, Radiology, Paris, France, [4]University Hospital Essen, Diagnostic and Interventional Radiology and Neuroradiology, Essen, Germany, [5]Siena University Hospital, Neuroimaging and Neurointervention, Siena, Italy, [6]Centre Hospitalier Universitaire Vaudois (CHUV) and University of Lausanne, Radiology, Lausanne, Switzerland, [7]Amsterdam UMC, Ophthalmology, Amsterdam, Netherlands*

**Purpose/Objectives:** Modern conservative treatment regimens of retinoblastoma often consist of image-guided techniques for trans-catheter direct delivery of chemotherapeutic agents into the ophthalmic artery: selective intra-arterial chemotherapy (SIAC). However, SIAC also induces intra-ocular toxicities including vitreal hemorrhage (18.1 %), retinal detachment (19.3 %), optic nerve atrophy (3.4 %) and vascular ischemia or chorioretinal atrophy (6.2 %). These intra-ocular toxicities could result in slower eye growth. Thus, the purpose of this study was to quantitatively see if SIAC influenced eye volume when comparing it to retinoblastoma eyes not treated with SIAC and to healthy contralateral eyes.
**Methods and materials:** In this multicenter retrospective study, patients from the European Retinoblastoma Imaging Collaboration (ERIC) with post-treatment MR imaging with at least T2-weighted and T1-weighted images (before and after gadolinium administration) were included.
All eyes were divided into three groups:
1) healthy eyes (healthy),
2) retinoblastoma eyes which weren't treated with SIAC (Rb w/o SIAC) and
3) retinoblastoma eyes which were treated with SIAC (SIAC). To assess the difference of the eye volume after SIAC, delineation of the whole eye (sclera including all the other intra-ocular structures) was performed on T1-weighted gadolinium infused MR images and validated by expert radiologists. For the comparison of the median volume between each group the Mann–Whitney U test was used. Linear regression was used for volume to evaluate if volume correlated with age and Rb w/o SIAC and SIAC.
**Result:** 210 retinoblastoma patients from the ERIC group with post-treatment MR scans were included (female n=90[43 %]; median age for the healthy eyes was 28 months (Interquartile range (IQR) 14-43 months), Rb w/o SIAC eyes was 19 months (IQR 8-30 months) and for SIAC eyes it was 23 months (IQR 13-38 months). Figure 1 shows a boxplot with the median volumes of the different groups.

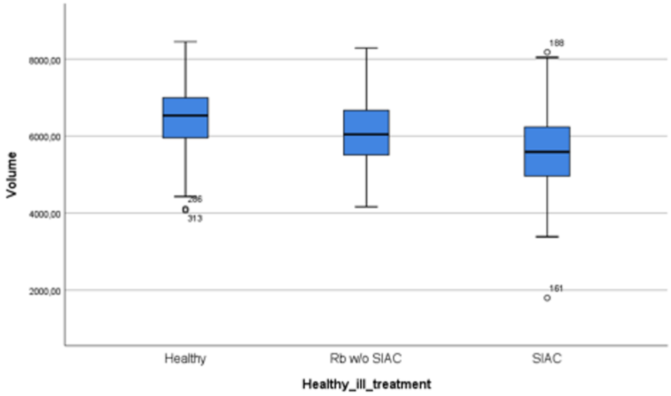

Boxplot with the median volumes of the three different groups.

The median volume of the healthy eyes was 6535 $mm^3$ (IQR 5947-6998 $mm^3$), for the Rb w/o SIAC eyes it was 6044 $mm^3$ (IQR 5461-6998 $mm^3$) and for SIAC eyes it was 5589 $mm^3$ (IQR 4954-6253 $mm^3$).
The median volumes of each group differed significantly (p<0.005).
In univariable linear regression of volume, Rb w/o SIAC vs. SIAC was a statistically significant predictor (p=0.001). Age was also a statistically significant predictor of volume in univariable linear regression (p=0.001) In a multivariable linear regression model both Rb w/o SIAC vs. SIAC and age were significant predictors with p-values<0.001.
**Conclusion:** This study demonstrates that SIAC has an negative effect on eye volume regardless of age, which could have implications for eye function and should be taken into consideration when treating with SIAC.

### Scientific Posters

### Giant cell granuloma and neurofibroma in the mandible in a patient with Neurofibromatosis type 1; A case presentation with dentomaxillofacial imaging aspects.

*Authors: O. Barut[1], M. Mukdad[2], K. Danielsson[3], P. E. Legrell[1], M. Sjöström[4]; [1]Västerbotten County Council, Department of Odontology, Oral and Maxillofacial Radiology, Umeå, Sweden, [2]Oral and Maxillofacial Surgery, Umeå University Hospital, Department of Odontology, Umeå, Sweden, [3]Umeå University, Department of Odontology, Orofacial Medicine, Umeå, Sweden, [4]Umeå University, Department of Odontology, Oral and Maxillofacial Surgery, Umeå, Sweden*

**Purpose/Objectives:** Giant cell granuloma (GCG) has been reported in the facial skeleton in people with Neurofibromatosis type 1 (NF1). The purpose of this case presentation is to raise the question if it might be beneficial to include the total facial skeleton in the follow-up imaging of patients with NF1 complications in the brain.
**Methods and materials:** A 29-year-old woman with NF1 is being followed up for her multiple neurofibromas on the skin and focal areas of signal intensity in the brain. A referral by the dermatologist was sent to the Orofacial Medicine Clinic for examination of the patient's complaints about tenderness, a bluish gingiva and mobile teeth in the anterior mandible. Cone beam computed tomography performed at the Oral and Maxillofacial Radiology Clinic revealed two separate lesions and extensive tooth resorptions in the anterior mandible. A review of the mandible in the multiplanar reconstructions of the patient's brain magnetic resonance imaging (MRI) disclosed the development of these lesions over time. A lesion that caused tooth resorptions had occurred sometime in the last two years and showed a rapid volume progression. The other lesion was unchanged during the last three years. Surgical removal of both lesions was performed by the oral and maxillofacial surgeon. Histopathological examination revealed GCG for the lesion with rapid progression and neurofibroma for the lesion without progression.
**Result:** A conservative treatment strategy with extirpation and short interval follow-up has been performed. At the one-year follow-up, clinical and radiological examination was uneventful with signs of bone healing. Four months later the patient experienced symptoms in the area for the GCG. The radiographic examination indicates recurrence of the pathology which can result in a more radical surgery, with resection, alveolar bone reconstruction and prosthetic replacement of anterior teeth.
**Conclusion:** This case presents two different pathologies; GCG and neurofibroma in the mandible of a patient with NF1 followed over time with MRI for the brain involvement. We conclude that it may be beneficial to include and evaluate the total facial skeleton in the imaging follow-ups of patients with NF1 complications in the brain, to identify any pathologies in the facial skeleton in early stages.

### Haller's cells features in a sample of Polish population – a panoramic radiography study

*Authors: I. Rozylo-Kalinowska[1], M. Piskórz[1], K. Futyma-Gabka[1], A. Manaj[1], A. Osiak[1], K. Orhan[1, 2]; [1]Medical University of Lublin, Department of Dental and Maxillofacial Radiodiagnostics, Lublin, Poland, [2]Ankara University, Faculty of Dentistry, Ankara, Turkey*

**Purpose/Objectives:** To characterize features of Haller's cells in a sample of the Polish population on the basis of panoramic radiographs.
**Methods and materials:** The study consisted of 467 panoramic radiographs including patients of both genders (303 females, 164 males), aged 17-23 years with a mean 20.2 years. All radiographs have been evaluated for Haller's cells' presence, shape, number and location with gender predilection. All the X-rays were taken due to clinical indications and not for the purpose of the study.
**Result:** Haller's cells were found in 72 cases, which comprised 15.4 % of the studied group, with a slightly higher prevalence in females (17.82 % in females and 10.97 % in males). The most predominant shape was oval. Unilateral distribution of the cells outnumbered bilateral variants. One to three Haller's cells were found on one side in the own material.
**Conclusion:** Panoramic radiograph revealed relatively low prevalence of Haller's cells in a sample of the Polish population. It is possible to assess the shape and determine the number of these cells, which are located more often unilaterally, with a slightly higher prevalence in females.

### High frequency intraoral ultrasound of the normal anatomy of the tongue: a preliminary study

*Authors: F. Borda[1], S. Caprioli[2], J. Ferro[3], A. Casaleggio[4], M. Fiannacca[1], G. Cittadini[5]; [1]University of Genova, DISSAL, Genova, Italy, [2]University of Genova, DIMI, Genova, Italy, [3]University of Genova, DISC, Genova, Italy, [4]IRCCS Ospedale Policlinico San Martino, Radiologia Interventistica, Genova, Italy, [5]IRCCS Ospedale Policlinico San Martino, Radiologia Generale, Genova, Italy*

**Purpose/Objectives:** High frequency intraoral ultrasound (IOUS) is a relatively new and promising technique for the preoperative staging of oral squamous cell carcinoma (OSCC), even if currently not routinely performed, partly due to a poor knowledge of the ultrasound anatomy of the tongue itself, partly for the high technological standards required. However, IOUS can be considered as a valid alternative to magnetic resonance imaging (MRI) for the staging of early tongue cancer. Thus, the aim of this work is to systematically study and describe the sonoanatomy of the oral tongue and to describe a scanning technique for a solid assessment.
**Methods and materials:** Twenty (20) healthy volunteers underwent IOUS examination using a high frequency, 8 mm footprint 22-8 MHz hockey stick probe. The tongue was scanned on both long and short axis, from dorsal, ventral and lateral surface. Superficial epithelial and subepithelial layer thickness were reported and the appearance, organization and thickness of intrinsic tongue muscles were described.
**Result:** High resolution IOUS allowed a reliable examination of tongue anatomy in all 20 healthy volunteers. A methodical approach was established in order to define the superficial layers and the deeper intrinsic and extrinsic muscles of healthy tongue.
**Conclusion:** Interest in high frequency IOUS of the tongue has been progressively increasing over time. The knowledge of the complex anatomy of the tongue and its ultrasound appearance is a fundamental requirement for the reliable and reproducible IOUS examination of tongue cancer. A standardized scanning technique can open new frontiers as, for instance, discriminating invasive and non-invasive mucosal lesions or differentiating benign or malignant submucosal neoplasm.

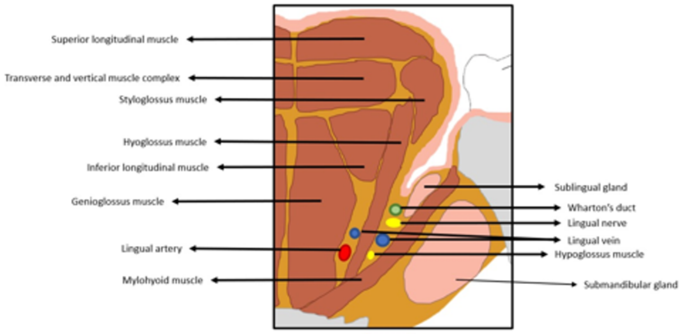

Tongue anatomical scheme

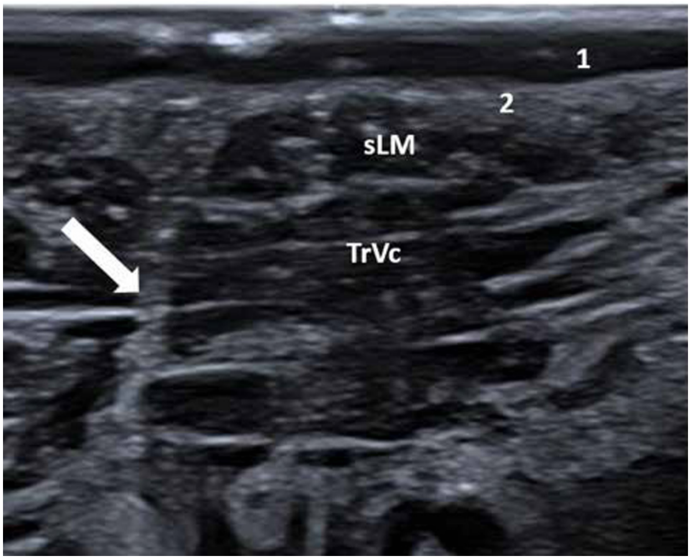

Coronal sonoanatomy of the tongue: 1: epithelial layer; 2: subepithelial layer; sLM: superior longitudinal muscle; TrVc: transverse and vertical muscle complex; Solid arrow: lingual septum

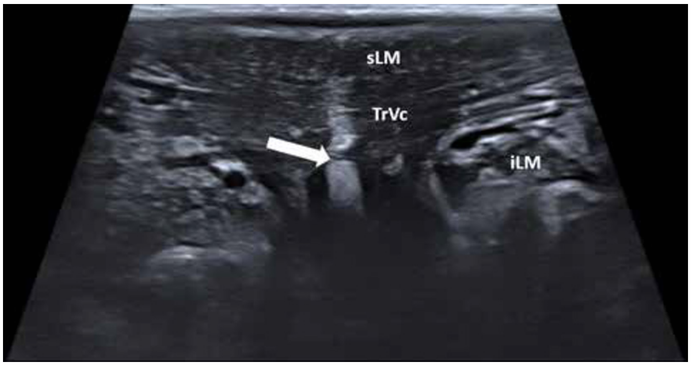

Extended FOV coronal sonoanatomy of the tongue: sLM: superior longitudinal muscle; TrVc transverse and vertical muscle complex; iLM inferior longitudinal muscle; Solid arrow: lingual septum

### Imaging role of the residual/recurrent pleomorphic adenoma

*Authors: S. Siurana[1], C. Bescos[2], L. Fite[2], M. Alberola[3], A. Rovira[1]; [1]Hospital Vall D'Hebron, Neuroradiology, Barcelona, Spain, [2]Hospital Vall D'Hebron, Maxillofacial Surgery, Barcelona, Spain, [3]Hospital Vall D'Hebron, Pathological anatomy, Barcelona, Spain*

**Purpose/Objectives:** Pleomorphic adenoma is the most common benign parotid gland tumor. Recurrence has been associated with pathology-related and surgery-related variables, tumor size and patient age. Malignant degeneration has been reported to be associated with a long duration of the tumor and with recurrent tumors. In many cases, initial diagnosis and treatment are conducted at different hospitals, so imaging/surgical records may not be available for radiological comparisons.

- To review our institutional experience with imaging of recurrent pleomorphic adenoma in the last ten years.
- To establish risks of secondary malignant transformation

**Methods and materials:** Retrospective imaging and chart reviews of 19 patients (10 males, 9 females; aged between 15–69 years) with recurrent pleomorphic adenoma of the parotid gland.
MR images of treated patients were reviewed as to the available sequences and location, multiplicity, signal intensity, cellularity and enhancement pattern
**Result:** Solitary recurrent lesion was seen in 5 patients and multiple in 14 patients.
The majority of recurrences were confined to the parotid space but in 6/19 cases they were located distant from the operative bed.
The volume of the recurrent tumor was ≤ 10 ml in 13, 10–20 mm in 2, 20–30 mm in 1, and ≥ 30 mm in T2 fat-saturation easily identifies most of recurrent lesion.
63 % of the lesion showed high T2 signal and high ADC.
Of the 7 lesions with ADC < 1.3, malignant transformation was identified in 3.
**Conclusion:** Understanding the diversity of radiological features of recurrent pleomorphic adenoma, using conventional and functional MR images, improves the radiologist's ability to make correct diagnoses.
Given the slow growth of residual lesions, and low risk of secondary malignant transformation MRI follow-up after all first recurrences, can help identify a patient's individual growth rate and can avoid a need for more extensive surgery.

### Post-irradiated carotid blowout syndrome in patients with head and neck malignancy – a report of 2 cases

*Authors: A. Knurowska[1], E. Pawłowska[2], I. Lapinska[3], B. Bascik[4], T. Nowicki[1]; [1]Medical University of Gdansk, 2nd Department of Radiology, Gdansk, Poland, [2]Medical University of Gdansk, Department of Oncology & Radiotherapy, Gdansk, Poland, [3]Medical University of Gdansk, Department of Otolaryngology, Gdansk, Poland, [4]Medical University of Gdansk, Department of Radiology, Gdansk, Poland*

**Purpose/Objectives:** To underline the importance of frequent surveillance and careful evaluation of carotid artery in patients with deep spaces necrosis after radiotherapy.
**Methods and materials:** We described two cases of carotid blowout syndrome (CBS) that occurred in patients with pharyngeal carcinoma after radiotherapy (RTx).
First patient, 43-year-old woman, was diagnosed with oropharyngeal carcinoma 6 month ago and treated with radical RTx (66Gy/33fr) and chemotherapy. In check-up MRI there was necrotic lesion with the exposure of the right ICA, without abnormalities within the arterial lumen. Finding was immediately reported to the referring physician. Patient was admitted to the emergency department 18 days after that with oral bleeding and hematemesis. Clinical examination at the admission revealed a necrotic lesion in the lateral wall of the oropharynx with active bleeding. Angiogram showed rupture of the right ICA, that was successfully repaired with stent grafts.
Second patient, a 44-year-old male, was admitted to the otolaryngological department a day after diagnosis of threatened CBS in check-up MRI. He was diagnosed with nasopharyngeal cancer (cT1N0M0) 3 years ago and treated with radical RTx (66Gy/30fr) and proton therapy after local recurrence. 12 days earlier he reported to the emergency department with oral bleeding, which resolved spontaneously. At the admission to the otolaryngological department, vessel wall irregularities in the right ICA and accompanying pseudoaneurysm with a diameter of 3mm were detected. At night after admission patient had oropharyngeal haemorrhage. During emergency endovascular treatment a stent graft was implanted.
**Result:** In the first case, necrosis of irradiated region occurred 6 month after treatment, whereas in the second case 1 year after proton therapy. First patient experienced oropharyngeal haemorrhage after 12 days since diagnosis of threatening CBS and the second patient just after one day. CBS usually develops after radiation with more than 80Gy.
**Conclusion:** CBS is a life-threatening complication of radiotherapy. In case of post-radiation necrosis in the deep spaces of the neck, carotid arteries must be carefully assessed. Difficult to predict progression of the disease should lead to more frequent imaging. In the event of any abnormalities of carotid arteries, preventive stent graft should be considered.

### The impact of reduced general bone mineral density on cortical bone of the edentulous mandible

*Authors: L. Jakaite[1], A. Beibakova[1], L. Neimane[2], A. Slaidina[3], O. Radzinš[4], I. Namatevs[5], K. Sudars[5]; [1]Riga Stradins University, Faculty of Dentistry, Riga, Latvia, [2]Riga Stradins University, Department of Conservative Dentistry and Oral Health, Riga, Latvia, [3]Riga Stradins University, Department of Prosthodontics, Riga, Latvia, [4]Riga Stradins University Institute of Stomatology, Riga, Latvia, [5]Institute of Electronics and Computing Science, Riga, Latvia*

**Purpose/Objectives:** The aim of this study was to detect impact of reduced general bone mineral density (BMD) on mandibular cortical bone thickness in edentulous postmenopausal females.
**Methods and materials:** In the present study 64 edentulous females were included, aged 54-87 years (mean age 70.4 ± 8.4y) who undertook cone beam computed tomography (CBCT) investigation (Next generation i-CAT) for implant planning.
Both femoral neck and lumbar spine BMD measurements were done by dual energy X-ray absorptiometry (DXA). The worst T-score reading from both was included. Patients were divided into 3 groups according to DXA results: normal BMD (T-score ≥ -1.0), osteopenia (T-score < -1.0 to -2.5) and osteoporosis (T-score ≤ -2.5).
CBCT images were analyzed with OnDemand3D software. In cross-sectional CBCT images, three areas of the mandible (lateral incisor, first premolar, first molar) were selected to determine vestibular and lingual cortical bone thickness. In the mental foramen region inferior cortical width of the mandible was measured.
To detect the differences between groups One-way ANOVA was used.
**Result:** Based on the DXA results, patients were stratified into 3 groups: normal BMD -18 (mean age 70.39±9.3y), osteopenia- 28 (mean age 70.29±8.23y) and 18 (mean age 70.56±8.2y) had osteoporosis (p=0.995).
The vestibular cortical bone width at incisive and premolar regions in osteoporosis and osteopenia groups was found to be less than in normal BMD group: osteoporosis (1.26 ± 0.32 mm), osteopenia (1.36±0.39 mm), normal BMD (1.79±0.59 mm); p=0.001; osteoporosis (1.40±0.38 mm), osteopenia (1.42±0.38 mm), normal BMD (1.76±0.48 mm), p=0.013.
Osteoporosis group also showed reduced inferior cortical bone thickness in the region of mental foramina: osteoporosis (2.63±0.73 mm), osteopenia (3.38±1.17 mm), normal BMD (3.09±0.72 mm), p=0.039.
There was no statically significant difference between the groups according to vestibular cortical bone thickness in molar region and lingual cortical bone thickness in all regions of mandible.
**Conclusion:** Postmenopausal females with reduced BMD showed reduced cortical bone thickness in the edentulous mandible.
**Funding:** This work was supported by the “Fundamental and Applied Research Projects”, grant number: lzp-2021/1-0031

### The relationship between bone mineral density and grey value measurements of jaw bones in postmenopausal females

*Authors: A. Beibakova[1], L. Jakaite[1], A. Slaidina[2], L. Neimane[3], O. Radzinš[4], I. Namatevs[5], K. Sudars[5]; [1]Riga Stradins University, Faculty of Dentistry, Riga, Latvia, [2]Riga Stradins University, Department of Prosthodontics, Riga, Latvia, [3]Riga Stradins University, Department of Conservative Dentistry and Oral Health, Riga, Latvia, [4]Riga Stradins University Institute of Stomatology, Riga, Latvia, [5]Institute of Electronics and Computing Science, Riga, Latvia*

**Purpose/Objectives:** The aim was to determine relation between general bone mineral density (BMD) to grey values (GVs) found using cone beam computed tomography (CBCT) in jaw bones of postmenopausal females.
**Methods and materials:** In the present study 64 edentulous females aged 54-87 years (mean age 70.4±8.4y) who underwent CBCT (Next-generation i-CAT, Kavo, Germany) examinations due to dental implant planning were included.
Bone mineral density measurements of lumbar spine and both femoral necks by dual energy X-ray absorptiometry (DXA) (Lunar DEXA DPX-NT, GE Medical Systems) were made. The worst T-score reading from both were taken into account. Based on DXA results patients were divided into 3 groups: normal BMD (T-score ≥ -1.0), osteopenia (T-score <-1.0 to -2.5) and osteoporosis (T-score ≤ -2.5).
CBCT images were analysed with OnDemand3D (Cybermed Inc., Korea) software. In cross-sectional CBCT images, three different areas of the mandible (lateral incisor, first premolar, first molar) and the region of tuber maxillae were selected to determine the average GVs of the jaws. Measurements were made with a 10 x 20 mm region of interest (ROI) in the middle of relevant cross-sectional images and were realized by two independent observers, each made measurements twice at a two-week interval.
The difference between groups was evaluated by One-way ANOVA with Bonferroni correction. Pearson correlation was used to determine a correlation between GVs and age. Measurement agreement was determined by Cronbach’s alpha test.
**Result:** There were 18 patients with normal BMD (mean age 70.39±9.3y), 28 patients with osteopenia (mean age 70.29±8.2y) and 18 patients with osteoporosis (mean age 70.56±8.2y). The age differences between groups were not statistically significant (p=0.995).
The osteoporosis group showed the lowest GVs compared to other groups, however, no statistically significant difference was found. A weak negative correlation was found between age and the first molar region measurements (r=-0.289, p=0.021).
Intraobserver and interobserver agreements were from acceptable to excellent ($0.68 \leq \alpha \leq 0.91$).
**Conclusion:** There were found no relationship between BMD and GVs of jaw bones in postmenopausal females.
This work was supported by the “Fundamental and Applied Research Projects”, grant number: lzp-2021/1-0031

### Usefulness of magnetic resonance imaging in the diagnosis of Parsonage-Turner syndrome: a single center study

*Authors: A. Napolitano[1], E. Contrino[2], C. Parisi[3], M. Cadioli[4], R. Capra[5], A. Lo Casto[2], S. Gerevini[1]; [1]Papa Giovanni XXIII Hospital, Department of Neuroradiology, Bergamo, Italy, [2]University Hospital of Palermo, Department of Biomedicine, Neuroscience and Advanced Diagnostics (BIND), Palermo, Italy, [3]GE Health Care, Palermo, Italy, [4]Philips Medical System, Milano, Italy, [5]Spedali Civili di Brescia, Department of Neurology, Brescia, Italy*

**Purpose/Objectives:** Parsonage-Turner Syndrome (PTS), also known as neuralgic amyotrophy, is a rare syndrome involving the peripheral nervous system, particularly the brachial plexus. The aetiology is unclear, but it is often associated with antecedent clinical events such as a recent viral infection or active immunization. The typical onset is a sudden pain in the shoulder, at night or on awakening, which is followed by progressive weakness of the upper limb and paraesthesia. Correct diagnosis isn't always straightforward. The aim of our study is to show MRI usefulness in the diagnosis of PTS, and describe pattern and characteristics in a group of patients with PTS.
**Methods and materials:** Consecutive patients referred to our center between October 2019 and February 2022 for suspected diagnosis of Parsonage-Turner were retrospectively included. Medical history, laboratory data and electrophysiological data were collected. All patient underwent MRI on a 3T system with a specific protocol for brachial plexus evaluation, including T1 and T2-weighted sequence, T2-weighted with fat suppression, T1-weighted contrast enhanced with and without fat suppression and high-resolution volumetric neurographic sequence (3D-STIR Nerve) after contrast injection.
**Result:** Of the 20 patients included (19M:1F, mean age: 50.75; range: 17-76 years), 14 showed unilateral symptoms (left 65 %), characterized in most cases by a typical onset, with pain in the scapular region and subsequent hyposthenia in the upper limb of variable degree. In almost the totality of cases, EMG findings reflected the distribution of alterations on imaging. MRI detected unilateral brachial plexus alteration in 14 patients. The alteration was assessed as T2-STIR hyperintensity and diffuse thickening. Enhancement of affected root was seen in one case. The most affected roots were C6 and C7 (80 % and 60 % respectively); some cases showed involvement of C8. Respectively, the most affected trunk and chord were the superior (60 %) and posterior (25 %). Involvement of the terminal branches of the brachial plexus (axillary and radial) was observed in two patients.
In regards to muscle involvement, trophism and signal were evaluated, with findings suggestive of oedema in 13 (65 %) patients as for acute-subacute denervation; in 10 cases (50 %) it was also associated with hypotrophy and adipose muscle fibers infiltration (chronic denervation changes). The most affected muscles were the supraspinatus (80%) followed by the infraspinatus (60 %) and deltoid (60 %).

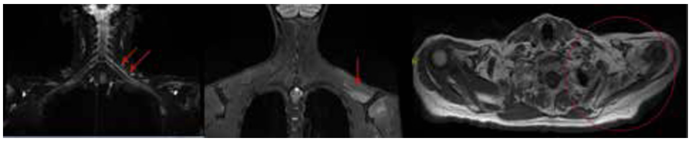

3DSTIR-nerve sequence shows thickening of nerve roots (in particular C6) with associated hyperintensity; coronal STIR shows heterogeneous oedema of the left supraspinatus and subspinatus muscles compatible with denervative oedema. In axial T1 mild hypotrophy of these muscle can be seen as for late changes of denervation.

**Conclusion:** MRI has a promising role for supporting the diagnosis in cases of suspected Parsonage-Turner. Furthermore, it allows an overall assessment of nerve and muscle involvement with possible implications for treatment and prognosis.

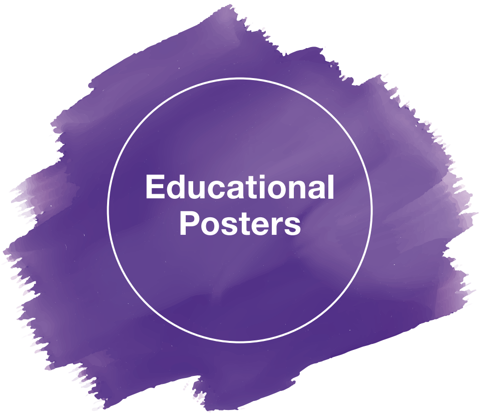

### Suggestion of optimal magnetic resonance imaging protocol for evaluation of hyaluronic acid filler deposits

*Authors: M. Kozeriew[1], M. Grzywinska[2], T. Nowicki[3]; [1]Medical University of Gdansk, Faculty of Medicine, Gdansk, Poland, [2]Medical University of Gdansk, Department of Physiology, Faculty of Medicine, Gdansk, Poland, [3]Medical University of Gdansk, 2nd Department of Radiology, Faculty of Health Sciences, Gdansk, Poland*

**Learning objectives:**
To present images of hyaluronic acid deposits in different MR sequences (TSE, STIR, TIRM, TSE DIXON, SPACE, CISS) with various parameters.
**Background:** In literature there is no consensus for imaging of facial fillers. We observe a drastic rise in popularity of hyaluronic acid injections – 337 % from year 2007 to 2020 only in US. Due to this phenomenon number of incidentally found filler deposits during routine radiologic examinations increases, as well as adverse reactions with the need for detailed determination of complications degree. Deposits with nonspecific image or in nonspecific locations, without history of previous injection can become a diagnostic challenge resulting in unnecessary biopsies or surgical treatment.
**Imaging findings and procedure details:** Patients with a well-known history of hyaluronic acid injections in different facial areas underwent head MRI. Presented images were acquired in different sequences (TSE, STIR, TIRM, TSE DIXON, SPACE, CISS) in different planes, with use of different matrix, flip angle, bandwidth, turbo factor, number of repetitions, with and without parallel imaging. With each image SNR and acquisition time is shown.
**Conclusion:** Magnetic resonance can accurately image the location of the hyaluronic acid deposits, but selecting the optimal protocol is important to obtain the balance between highest possible image quality and cost efficiency. Selecting the optimal protocol can be considerable help in ambiguous cases.

### A Pictorial Review of Perineural Spread of Head and Neck Malignancy

*Authors: I. Irvine, R. Hayden, G. Allen, P. Cooper, A. Francis, M. Farrell; University Hospital Waterford, Radiology, Waterford, Ireland*

**Learning objectives**
An overview of the clinical features and implications of perineural tumour spread.
The important imaging findings to look for in perineural spread.
**Background:** Perineural spread is the direct extension of a primary tumour along a nerve. It is a form of local invasion that is well described in head and neck malignancies. It is classically associated with adenoid cystic carcinoma, in which it occurs in up to 60 % of cases. However, in clinical practice it is most commonly seen with squamous cell carcinoma due to its far higher incidence, and also occurs with many other malignancies.
Perineural spread typically presents with cranial nerve palsy or neuralgia but can be asymptomatic in 30-45 % of cases. Any nerve can provide a route for disease spread, but the facial nerve and the maxillary and mandibular branches of the trigeminal nerve are most commonly affected. It is an important disease manifestation to recognize as it has implications for prognosis and treatment strategies.
**Imaging findings and procedure details:**
Imaging is best performed with post-contrast MRI but post-contrast CT can also be utilized. The major imaging features include nerve enlargement and enhancement, obliteration of perineural fat at the foraminal openings, and erosion or asymmetrical enlargement of the skull base foramina which can be very well appreciated on CT.
Nerve disease will result in muscle denervation, and radiologists should examine for muscular oedema and enhancement, and fatty atrophy in the chronic setting.
**Conclusion:** Radiologists play an important role in the diagnosis of this entity and in delineating the exact route of disease spread. Imaging findings can be subtle and therefore a comprehensive knowledge of both the anatomy and disease manifestations is required.

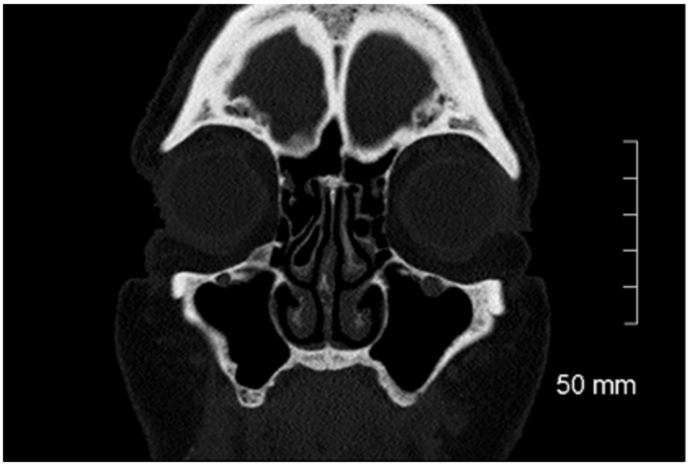

Coronal CT: Enlargement of the left infraorbital foramen

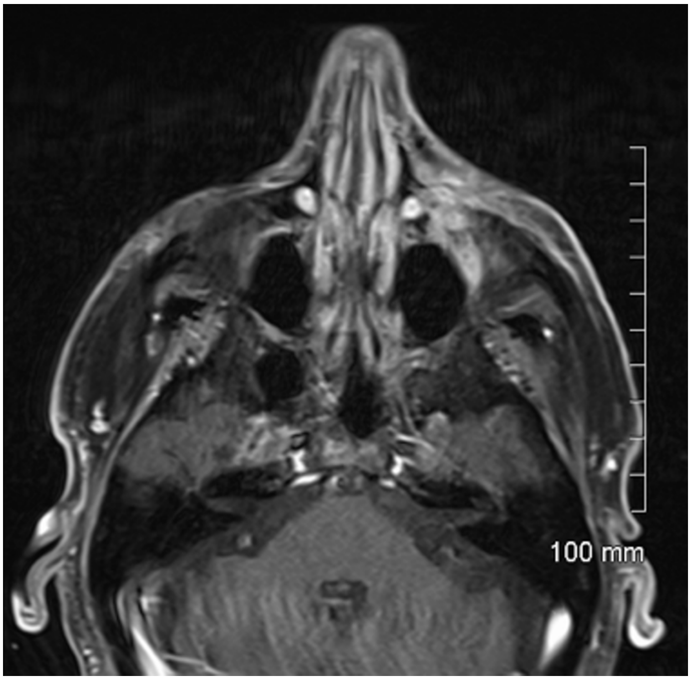

Axial post-contrast MRI: Enhancement and enlargement of the left infraorbital nerve

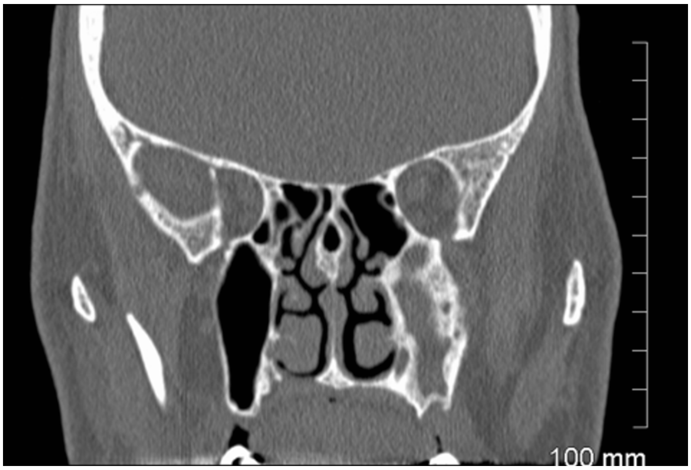

Axial post-contrast MRI: Enhancement and enlargement of the left infraorbital nerve

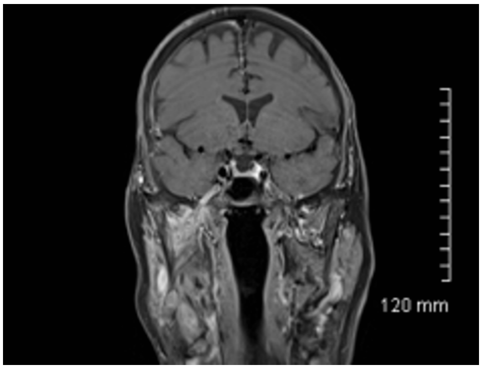

Coronal post-contrast MRI: Denervation enhancement of the right masticator muscles. Perineural spread via the mandibular branch of the right trigeminal nerve through foramen ovale.

### A real pain in the neck!

*Authors:* *M. Kumar, R. Rhys; Royal Glamorgan Hospital, Cwm Taf Morgannwg Health Board, Radiology, Cardiff, United Kingdom*

**Learning objectives:** Hyoid Bone Syndrome is a little-known entity which if recognized may be treated successfully. It should be considered in patients with symptoms of sharp, stabbing pain in the neck with focal tenderness at the body and greater cornu of the hyoid bone.
The aim of this poster is to highlight the clinical presentation of Hyoid Bone Syndrome including a case study showing both USS and MRI findings and successful treatment using steroid injection.
**Background:** The hyoid bone is a non-articulating U-shaped structure in the anterior neck which lies between the base of tongue and the thyroid cartilage. It is a site for insertion of numerous tendons and ligaments, which keeps the bone suspended, and facilitate movement in all four directions during respiration, swallowing and phonation.
Hyoid Bone Syndrome is synonymously used with ‘hyoid bone insertion tendinitis’ (1). The term tendinitis is largely replaced by tendinopathy, which is a clinical syndrome consisting of pain, tenderness and tendon swelling. Tendinopathy of the muscles that insert into the hyoid bone can lead to a range of symptoms such as stinging pain that is exacerbated by neck movements, respiration, swallowing and phonation. On clinical examination, there is focal tenderness at the greater cornu of the hyoid bone. As the clinical symptoms may be rather vague, the clinical workup for anterior neck pain may consist of a range of imaging modalities, such as a cervical spine X-ray to look for an occult fracture, USS to look for sialdenitis, and an MRI scan to look for Temporomandibular joint (TMJ) dysfunction.
**Imaging findings and procedure details:**
MRI is the most sensitive modality to diagnose Hyoid Bone Syndrome, the signs may be quite subtle. A careful search should be made for signs of acute inflammation in the tendinous attachments to the hyoid bone and marrow oedema in the hyoid bone. Tendon hyperaemia and subtle soft tissue inflammatory change in the soft tissues around the hyoid bone may be demonstrated on ultrasound. In patients who have previously undergone surgical procedures such as a hemi-hyoidectomy, histopathological surgical specimens have yielded results showing changes to support insertional tendinopathy (1).
Hyoid Bone Syndrome may be treated conservatively with topical or systemic NSAIDs. If these fail, localized steroid injections at the site of inflammation can be given. These have proven to be effective in our center.
**Conclusion:** Hyoid Bone Syndrome is an unfamiliar clinical entity and is often a diagnosis of exclusion. A careful history and an awareness of the subtle findings on MRI and US enable an early diagnosis. We describe a case of this syndrome and its successful treatment with targeted USS-guided steroid injection to alleviate symptoms.

### Anatomical variability of nasopalatine canal in Cone-Beam Computed Tomography

*Authors: I. Rozylo-Kalinowska[1], M. Piskórz[1], W. Kielt[1], J. Kozlowska[1], K. Orhan[1, 2]; [1]Medical University of Lublin, Department of Dental and Maxillofacial Radiodiagnostics, Lublin, Poland, [2]Ankara University, Faculty of Dentistry, Ankara, Turkey*

**Learning objectives:** To understand anatomical variability of nasopalatine canal (NPC) in Cone-Beam Computed Tomography (CBCT) studies.
**Background:** NPC connects the floor of the nasal cavity with the anterior part of the hard palate. Variability of NPC dimensions and the thickness of maxillary bone anterior to NPC are related to age, gender and ethnicity. Also the shape, position and number of NPC nasal floor openings vary considerably. CBCT is more and more often used in dentomaxillofacial imaging and knowledge on potential influence of NPC anatomy on diagnosis and treatment planning is crucial especially when implant placement, treatment of nasopalatine cyst and nasopalatine nerve block are to be performed.
**Imaging findings and procedure details:** One hundred consecutive CBCT scans of 55 females and 45 males, aged 20-30 years were evaluated for features such as:. shape, length and width in the narrowest part of the canal, antero-posterior measurement of nasal and palatal openings and medio-lateral diameter of incisive opening, number of nasal openings and division of NPC, the presence of additional foramina to the incisive canal and the thickness of maxillary bone anterior to the NPC. Patient gender had a significant effect on all NPC parameters with the exception of shape of the canal, foramina of Stenson's and canalis sinusous presence. Mean values in males were higher. The most common shape was cylindrical and the rarest – banana-like. Single NPC canal was the most prevalent and more often observed in females.
**Conclusion:** Although single, cylindrical NPC is the most prevalent presentation in the analyzed CBCT sample, knowledge on anatomic variations of NPC is important in diagnostics and treatment planning.

### Can you see it crystal clear? A systematic review of imaging evaluation of the crystalline lens.

*Authors: T. Chamel[1], F. Baccega[2], V. Castanheira[2], O. Saito[1], E. Gebrim[1], R. Gomes[1]; [1]Hospital das Clínicas da Faculdade de Medicina da Universidade de São Paulo, Radiology, São Paulo, Brazil, [2]Hospital das Clínicas da Faculdade de Medicina da Universidade de São Paulo, Ophthalmology, São Paulo, Brazil*

**Learning objectives:**
- To review the anatomy and physiology of the crystalline lens, correlated to its normal appearance in ultrasound, computed tomography and magnetic resonance imaging.
- To refine the radiologic propaedeutics of the lens through a systematic evaluation.
- To be familiarized with imaging findings of some of the most relevant lens disorders.

**Background:** Clinical evaluation of the crystalline lens generally includes visual acuity examination and slit-lamp biomicroscopy, eventually combined with ultrasound techniques (e.g. B-scan sonography, biomicroscopy ultrasound). Although ophthalmologists rarely rely on cross-sectional imaging for routine assessment of patients with lens disorders, computed tomography and magnetic resonance imaging gain importance when other methods are constrained by technical limitations, like in the setting of traumatic orbital injury or mean opacification of the anterior segment of the eyeball. In addition, it is pivotal to raise alert when important alterations of the posterior chamber of the globe, where lies the lens, appear as incidental findings in scans obtained for other purposes. Therefore, the ability to accurately recognize the various imaging manifestations of lens disorders is critical for radiologists to avoid misdiagnosis.
**Imaging findings and procedure details:** Anatomy of the lens and its spatial relations within the eyeball, as well as the lens's physiology and its important pathophysiological implications, are reviewed through schematic graphics, and examples of normal radiological appearance are shown. A systematic imaging evaluation of the lens is proposed, taking into account three main features: morphology, position and texture. A selection of lens disorders and clinical settings, including age related cataract, cataract surgery and intraocular lens implants, trauma, congenital and syndromic disorders, and miscellaneous disorders with lens implications are discussed on the basis of a case compilation from our institution.
**Conclusion:** Although relatively neglected on cross-sectional imaging, the crystalline lens holds valuable information concerning not only its own condition, but also disorders that affect the whole eyeball and its surroundings. Computed tomography and magnetic resonance imaging appearance of the lens should be promptly assessed together with other ocular imaging findings, and wisely used towards a sharp diagnosis, optimizing patient care.

### Dual energy CT in head and neck: what relevant information can we obtain?

*Authors: L. Oleaga[1], S. Medrano[1], J. Berenguer[1], A. Muxi[2], I. Vilaseca[3], I. Valduvieco[4], N. Baste[5], P. Castillo[6]; [1]Hospital Clinic, Radiology, Barcelona, Spain, [2]Hospital Clinic, Nuclear Medicine, Barcelona, Spain, [3]Hospital Clinic, Otorhinolaryngology, Barcelona, Spain, [4]Hospital Clinic, Radiotherapy, Barcelona, Spain, [5]Hospital Clinic, Oncology, Barcelona, Spain, [6]Hospital Clinic, Pathology, Barcelona, Spain*

**Learning objectives:**
- To show the utility of Dual energy CT in head and neck pathology
- To illustrate a distinct spectrum of head and neck benign and malignant processes
- To highlight the potential advantages of dual energy CT over standard contrast enhanced CT in head and neck lesions

**Background:** The concept underlying dual-energy CT is the difference in the x-ray absorption of different materials as a function of x-ray energy, particularly for materials with a high atomic number Z such as iodine. By using two different energies, material-specific images can be obtained postprocessing the acquired data, to generate different images such as virtual unenhanced, virtual monoenergetic, and material-specific iodine images.
Dual energy Computed Tomography (DECT) can play an important role in the evaluation of head and neck pathology. The images generated provide anatomic and functional information, improving lesion detection and providing quantitative calculation of the degree of enhancement that may aid to further characterize benign or malignant lesions as well as posttreatment changes. Virtual non-contrast images can be calculated from the contrast-enhanced acquisition with the consequent benefit of radiation dose reduction.
**Imaging findings and procedure details:**
DECT can accurately characterize head and neck lesions. Virtual contrast-fee maps and iodine quantification can help characterizing head and neck lesions and differentiating posttreatment changes from recurrences in patients with malignant tumours.
We describe and illustrate imaging findings in a variety of patients with benign and malignant head and neck lesions.
**Conclusion:** DECT provides morphologic and quantitative information regarding tissue composition.
The possibility of obtaining material-specific images from a contrast-enhanced DECT allow radiation dose reduction and quantification of iodine to better characterize head and neck lesions

### Fibrosseous Maxillofacial Lesions

*Author: M. Milicevic; University Hospital of Liege, Radiology, Liège, Belgium*

**Learning objectives:**
We suggest a proposal for approaching the differential diagnosis of maxillofacial fibrosseous lesions.
Demographic data, clinical presentation, imaging findings and pathological results are discussed.
**Background:** Fibrosseous maxillofacial lesions are mostly asymptomatic incidental findings. Treatment is indicated only for cosmetic reasons or for infectious complications. In cranio-facial location, ossifying fibroma and fibrous dysplasia predominate, in maxillary location, ossifying fibroma, fibrous dysplasia, peri-apical and florid cementosseous dysplasias are most common findings. In differential diagnosis we must include other fibrosseous lesions, neoplasias and infectious diseases. The correct diagnosis is established by clinical signs and imaging findings. In rare cases, cross correlation with pathology remains unsatisfactory.
Before advent of CBCT, fibrosseous lesions of maxillofacial location were diagnosed incidentally by CT, MRI, or orthopantomogram. Special attention was paid to cases complicated by infection, bone enlargement or when cystic or malignant degeneration was suspected. Wide use of CBCT allowed for an earlier diagnosis of these pathologies and periodic monitoring of lesion extent and diagnosis of complications.
**Imaging findings and procedure details:** Diagnostic work-up and differential diagnoses are presented in two tables. Illustrative images are shown. Periapical and florid cementosseous dysplasias are of exclusive maxillary location. Differential diagnosis should include fibrous dysplasia, ossifying fibroma, Paget disease, apical osteitis, odontoma, osteomyelitis and cementoblastoma. Fibrous dysplasia and ossifying fibroma can be of facial or maxillary location. Differential diagnosis should consider osteosarcoma, osteoma, cementoblastoma, Paget disease, cementosseous dysplasia and osteomyelitis.
Lesion expansion, density values on CB(CT), marginal demarcation and mass effect are important descriptive lesional features. MRI or pathological assessment are rarely necessary for establishing diagnosis. Other associated pathologies such as Jaffe-Lichtenstein syndrome or McCune Albright syndrome in connection with fibrous dysplasia are of rare occurrence. In extended fibrous dysplasia, neurovascular conflicts can occur. A simple bone cyst or secondary infection may be seen in periapical and florid cementosseous dysplasia. Involvement of paranasal sinuses can result in mucocele or facial deformity.
**Conclusion:** CBCT, clinical presentation, demographic data and natural history are adequate for establishing the etiopathogenic diagnosis of fibrosseous lesions in majority of cases. Pathological confirmation is rarely required. Surgery is indicated for treatment of infection, cystic or sarcomatous degeneration or for cosmetic reasons.

### Head and Neck Myiasis: an Approach to the Main Imaging Findings

*Authors: R. Gomes, R. Andrade, M. Brandão, H. Tames, R. Murakoskhi, E. Gebrim; Universidade de São Paulo, Instituto de Radiologia, São Paulo, Brazil*

**Learning objectives:**
- Identify the typical imaging features that suggest the diagnosis of myiasis;
- Assemble some cases to illustrate the most often sites affected in the head and neck region and the importance of imaging methods to evaluate the extension and complications of disease;
- Alert the radiologists about the underlying causes of myiasis and the association with neoplasms.

**Background:** Myiasis is a dermatozoonosis caused by the dipterous larvae infestation, developing on devitalized tissues. This disease is more frequently found in tropical regions, and the main risk factors are associated with poor healthy and socioeconomic conditions.
Some of most often sites affected in the head and neck region are opening orifices, including ears, oral cavity, nose, paranasal sinuses and tracheostomy wounds, but lymph nodes and temporal bone also can be injured.
This education exhibit aims to demonstrate didactic cases with head and neck involvement in myiasis, illustrate the association with malignant neoplasms and the importance of their recognition. When clinically appropriate, biopsy of the wound site should be obtained.

**Imaging findings and procedure details:** The typical imaging features include:
- Inflammatory changes;
- Linear areas permeating the affected tissues that correspond the larvae;
- Tissue loss and local necrosis;
- Bone erosion/destruction.

**Conclusion:** Although the recognition of the disease is usually straightforward, the imaging appearance is important, mainly when associated with neoplastic lesions or other complications.

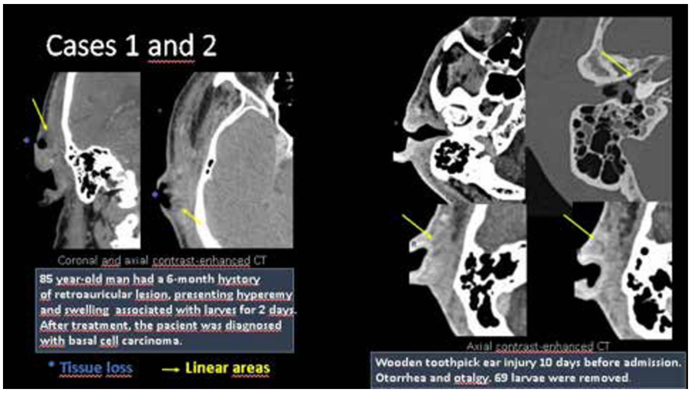

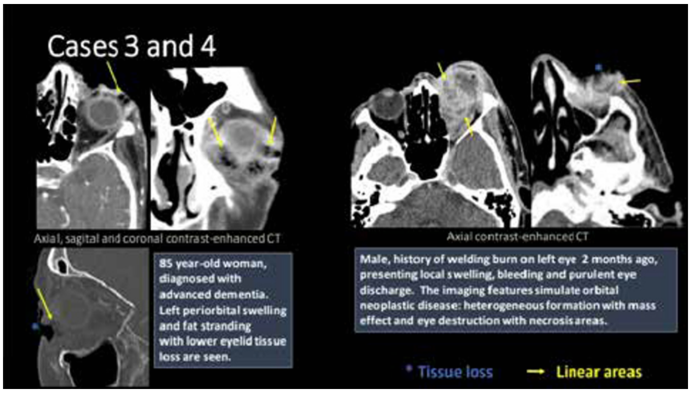

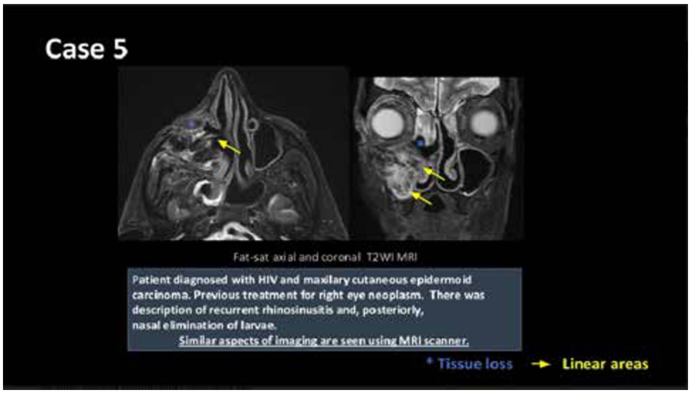

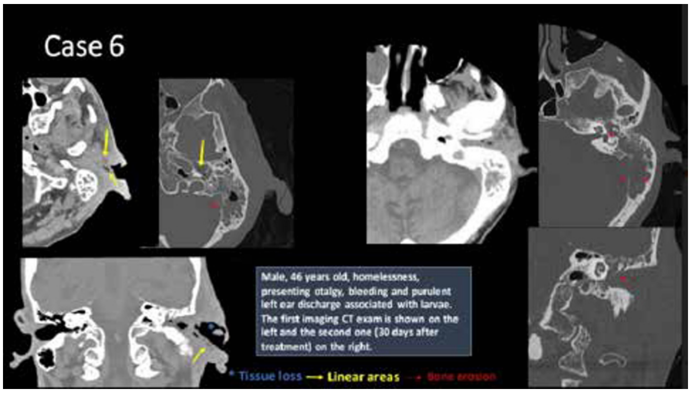

### Imaging findings in idiopathic resorption of mandibular condyle

*Authors: M. Prenc[1], T. Badel[2], D. Zadravec[1], M. Smoljan Basuga[1]; [1]UHC Sisters of Mercy, Department for Diagnostic and Interventional Radiology, Zagreb, Croatia, [2]School of Dental Medicine, University of Zagreb, Zagreb, Croatia*

**Learning objectives:** To present radiological characteristics of idiopathic condylar resorption (ICR) on different imaging modalities and to assess contribution of radiological manifestations in setting diagnosis, follow-up and managing treatment.

**Background:** Mandibular condyle is the process on the mandible that articulates with the disk of the temporo-mandibular joint (TMJ). It consists of the condylar process and head of the mandible. Shape and size of condyles differ among individuals as anatomical variations or as morphological changes in many diseases. ICR, also known as condylolysis or condylar atrophy is a rare progressive condition with unknown etiology that causes erosion of condylar head with consequent loss of condylar volume and alteration of condylar shape. It commonly occurs under the age of 20 years with 9:1 female-to-male frequency ratio and is almost always bilateral. First signs indicating ICR are retrognathism, decrease in posterior facial height, progressive malocclusion and symptoms of TMJ dysfunction. Most valuable imaging methods for diagnosis are cone-beam computed tomography (CBCT) and magnetic resonance imaging (MRI).

**Imaging findings and procedure details:** This case presents a 18-year follow-up of a 28-year-old female patient who initially complained about symptoms in the right TMJ that lasted for 2 months. On clinical examination patient reported right TMJ pain intensity 7 out of 10 at rest that escalated when opening mouth and radiated in right cheek and right ear with pain in the bilaminar zone and myofascial pain of the right masseter. Contacts between teeth were disturbed with signs of wear most prominent on anterior mandibular teeth. Radiological examination started with panoramic imaging and cephalogram that showed malocclusion with marked retrognathia. MRI proved anterior disc displacement without reduction of the right TMJ, which correlated with clinically observed clicking.

Patient underwent conservative therapy with occlusal splint. Despite therapy, follow-up examinations showed signs of progressive osteoarthritis that clinically manifested as crepitations. On panoramic imaging right condyle was significantly smaller than healthy left condyle, right mandibular ramus was shortened and mandible was clockwise rotated with consequently open bite. CBCT and MRI in more detail showed features of osteoarthritis with irregular cortical outline and marrow signal of volume-diminished condylar head. Additional laboratory and genetic studies were made and excluded other rheumatological or hereditary primary disease.

**Conclusion:** Pathogenesis and management of ICR are still poorly understood. Radiological findings are often suggestive for ICR and should be considered in diagnosis, stagging and treatment planning.

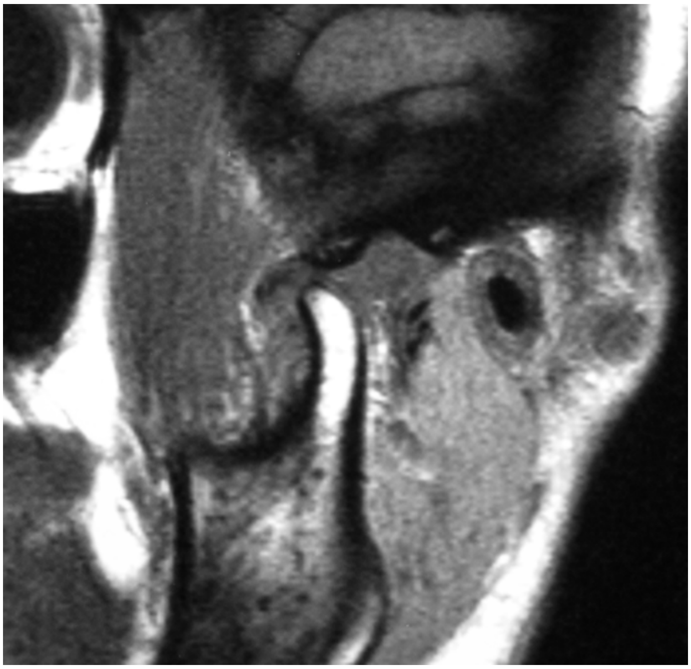

MRI of the right TMJ during open mouth shows anterior disc displacement without reduction

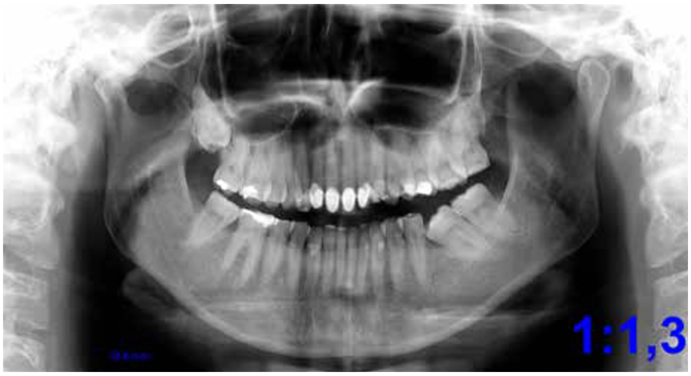

Panoramic imaging shows asymmetry with right condyle being smaller than left condyle

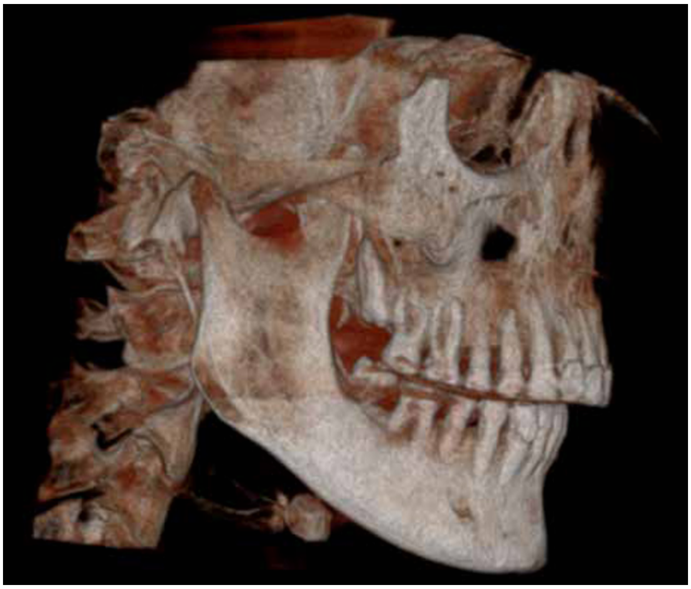

3D reconstruction of the right TMJ showing retrognathia, signs of osteoarthritis and changes of size and shape of the right condyle

### Imaging of Developmental Anomalies of the Orbit

*Authors:* *S. Hooker, B. Ozgen; University of Illinois at Chicago, Radiology, Chicago, United States*

**Learning objectives:** Congenital orbital anomalies are complex developmental disorders that usually require high resolution MR imaging for appropriate detailed evaluation. The developmental abnormalities detailed in this review will include anomalies of the eye such as anophthalmia, microphthalmia, cryptophthalmos, macrophthalmos, coloboma, staphyloma, morning glory disc anomaly, optic nerve hypoplasia and aplasia, persistent fetal vasculature, retinal dysplasia; as well as congenital orbital anomalies such as dermoid/epidermoid cyst, dacryocystocele, congenital fibrosis of the extraocular muscles.
**Background:** The orbital structures and especially the eyes are formed by a complex, integrated process and any error in this process can lead to a large spectrum of developmental anomalies of the eye or orbit.
While there might be external evidence of an abnormality in some lesions, the full extent of the malformation can be best assessed with imaging. Furthermore, imaging can also reveal anomalies in an externally normal appearing eye of a patient with a visual dysfunction.
The eye forms as an outgrowth from the anterolateral portion of the embryonic neural tube. Although the malformations of the eye can occur in isolation, they can accompany brain malformations or be a part of multisystem anomaly. Imaging is thus not only instrumental in correctly characterizing these lesions, for accurate diagnosis but also for detection of accompanying additional intracranial findings.
This exhibit will review the imaging findings of congenital orbital pathologies, with a brief overview of the potential clinical implications.
**Imaging findings and procedure details:** The imaging findings of various congenital orbital lesions will be reviewed with illustrative cases to demonstrate pertinent radiological findings.
**Conclusion:**
This exhibit will review the imaging findings of developmental anomalies of the orbit. These lesions can result in lifelong visual handicap, functional difficulties or cosmetic problems and their accurate diagnosis will guide appropriate patient management. Furthermore, as the developmental abnormalities of the anterior visual pathways can have accompanying intracranial or systemic problems, their accurate imaging diagnosis thus has even greater significance.

### MRI fistulography for branchial cleft sinus – Two case reports on a novel imaging technique

*Authors:* *S. Telli Erdogan, A. Inkiläinen, L. Flygare; Norrlands University Hospital, Radiology Department, Umeå, Sweden*

**Learning objectives:** To introduce a new technique when performing MRI fistulography for branchial cleft sinus imaging
**Background:** Branchial cleft anomalies are common cause for congenital neck masses, most often diagnosed in childhood. Treatment of choice is complete surgical excision. Due to their origin they are often located adjacent to vital neural and vascular structures in the neck. Various radiological methods for preoperative mapping have been described, including CT fistulography which has the major downside of radiation exposure to an often pediatric or adolescent population.
**Imaging findings and procedure details:** Two cases with a cutaneous sinus with purulent secretion were admitted to radiology unit for magnet resonance imaging (MRI). The first case was a 25-year-old man with a right sided cutaneous sinus. The orifice of the sinus was located in the supraclavicular region, along the border of the sternocleidomastoid muscle. The second case was a 10-year-old girl with an identical right sided sinus. Both sinus openings were cannulated and saline solution was injected using a thin plastic syringe. MRI scan was performed for confirmation and mapping of the sinus tract.
**Conclusion:** In the two presented cases MRI fistulography provided an excellent method for diagnosis and preoperative mapping of the branchial cleft sinus tract. MRI provides better soft tissue discrimination compared to CT and is a radiation free method. Injection of saline solution via the skin opening can also help discern branchial cleft sinuses from fistulae.

### MRI for Vestibular Schwannoma – when is Gadolinium required?

*Authors:* *D. Matthews[1], A. J. Lucarz[2]; [1]Royal Alexandra Hospital, NHS Greater Glasgow and Clyde, Clinical Radiology, Paisley, United Kingdom, [2]NHS Greater Glasgow and Clyde, Royal Alexandra Hospital, Clinical Radiology, Paisley, United Kingdom*

**Learning objectives:**
1. Audit the frequency of Gadolinium use in our Institution for the investigation and follow up of Vestibular Schwannoma.
2. Identify ways to reduce the amount of Gadolinium being administered.
3. Construct a new imaging pathway rationalizing the use of Gadolinium.

**Background:** Vestibular Schwannoma (VS) is the most common benign tumour encountered in the cerebellopontine angle/internal auditory canal. The diagnosis and follow up of these lesions is heavily dependent on MRI of the internal acoustic meatus (MRI IAM) as it provides high levels of diagnostic sensitivity and specificity.
The use of Gadolinium enhanced MRI IAM was anecdotally noted to be the default protocol used in our Institution, NHS Greater Glasgow & Clyde (Scotland), for the majority of VS imaging. The risk of allergic reaction, possibility of contrast accumulation within brain parenchyma and increased scan times led us to consider if we could reduce the amount of Gadolinium being used in these patients.
**Imaging findings and procedure details:** We set out to audit current practice in our Institution with the aim of mapping the imaging pathway currently being used for VS patients and identifying opportunities to rationalize and reduce the amount of Gadolinium being used.
A retrospective audit of the MRI sequences acquired in patients being investigated/followed up for VS across NHS Greater Glasgow & Clyde in 2020 and 2021 was completed. This confirmed a heavy dependence on Gadolinium enhanced imaging for screening, diagnostic and follow up studies.
A review of current literature demonstrated that there are non-contrast enhanced sequences that can be successfully used for treated VS follow up. In light of this, we have constructed a new pathway for VS patients that, whilst maintaining diagnostic accuracy, will greatly reduce the amount of contrast being used.
**Conclusion:** Gadolinium enhanced MRI sequences are well established in the imaging of Vestibular Schwannoma. Our project demonstrates that there is scope to rationalize and reduce the use of contrast by utilizing alternative, non-contrast enhanced, MRI sequences. We propose a new imaging pathway to allow a more strategic use of Gadolinium in the imaging of Vestibular Schwannoma.

### Necrotizing otitis externa: challenging diagnosis and follow-up imaging findings

*Authors:* *Y. Pekçevik, I. B. Arslan, I. Çukurova; Health Sciences University, Tepecik Training and Research Hospital, Radiology, Izmir, Turkey*

**Learning objectives:**
1. To review diagnostic clinical and imaging findings of the necrotizing otitis externa
2. To demonstrate key imaging features of the NEO for differential diagnosis
3. To show spread patterns and complications during the follow-up period

**Background:** Necrotising otitis externa (NOE) has clinical and radiological challenges at the time of diagnosis and during the follow-up period. It is a rare life-threatening disease with nonspecific symptoms. Several diseases can mimic SBO at imaging. A high index of clinical suspicion and appropriate imaging is required for diagnosis. The follow-up period is also challenging and early diagnosis of treatment response and complication is very important for patients' management. Magnetic resonance (MR) imaging of the skull base is superior for diagnosing the NEO and evaluating intracranial complications related to NEO.
We aim to present case-based clinical and imaging findings of the NEO, review differential diagnoses, and demonstrate spread patterns and complications
**Imaging findings and procedure details:** We reviewed clinical and imaging findings of 30 patients with NOE at the time of diagnosis and during follow-up. Key imaging findings for differential diagnosis, spread patterns of the disease, and complications were demonstrated.
**Conclusion:** NEO is an uncommon but life-threatening condition. Being familiar with imaging and clinical findings at the time of diagnosis and during follow-up helps early diagnosis of the NEO and recognizing complications.

### Pathology of the lacrimal gland: review of anatomy and imaging features on CT and multiparametric MRI

*Authors:* *S. Grauwels, P. de Graaf, M. De Win; Amsterdam University Medical Centers, Radiology & Nuclear medicine, Amsterdam, Netherlands*

**Learning objectives:**
- Anatomy of the lacrimal apparatus: lacrimal gland, and nasolacrimal drainage apparatus.
- Common pathologies throughout the apparatus and their respective imaging features.
- Considerations of the ophthalmologists and other surgical colleagues.

**Background:** The anatomy of the lacrimal apparatus, from the lacrimal gland to the nasolacrimal drainage apparatus is an area many radiologists are unfamiliar with, and which may be overlooked or misinterpreted in a routine search pattern.
There is a wide array of pathologies which can be present in this relatively small anatomic area, some of which can be clinically diagnosed and evaluated. However, in some situations imaging can be necessary, allowing the radiologist to provide important information for referring physician and help guide further treatment and planning.

**Imaging findings and procedure details:** This poster shall primarily focus on the CT and MRI imaging characteristics of anatomy and different pathologies. This includes infection/inflammation, benign and malignant tumors.
The additional value of multiparametric MRI techniques such as DWI (diffusion weighted imaging) and DCE (dynamic contrast enhancement) perfusion in further characterizing these lesions will also be discussed.
We will also emphasize what the ophthalmologist / orbital and oculoplastic surgeon wants to know from us.

Examples of the use of conventional and multiparametric MRI:

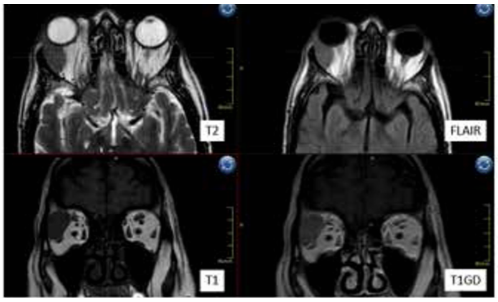

Example of multiparametric MRI1
M65 presenting with proptosis, having developed over one month. Without multiparametric MRI, the differential diagnosis consisted of: pseudotumor, mixed tumor lacrimal gland, lymphoma, vascular malformation of the cavernous type. With the use of multiparametric imaging, it was possible to narow the differential to most likely lymphoma, possibly pseudotumor.

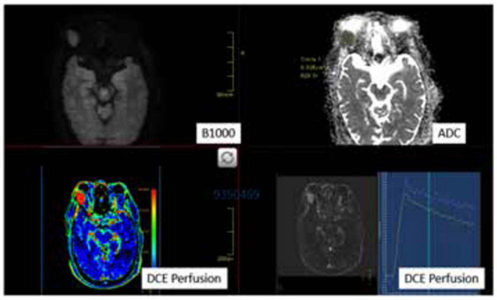

Example of multiparametric MRI1b
M65 presenting with proptosis, having developed over one month. Without multiparametric MRI, the differential diagnosis consisted of: pseudotumor, mixed tumor lacrimal gland, lymphoma, vascular malformation of the cavernous type. With the use of multiparametric imaging, it was possible to narow the differential to most likely lymphoma, possibly pseudotumor.

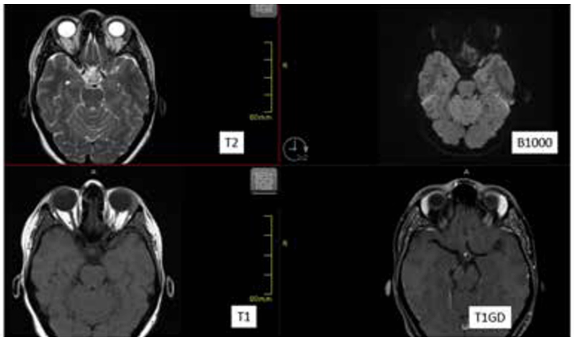

Bilateral disease with relevant multiparametric MRI
F28 with colitis ulcerosa presenting with a chronic painful left eye. MRI shows bilateral enlarged and enhancing lacrimal gland, with the differential diagnosis: M. Sjogren, sarcoidosis. The lack of diffusion restriction made lymphoma less likely.

Example of the use of characteristic CT findings to narrow the differential diagnosis:

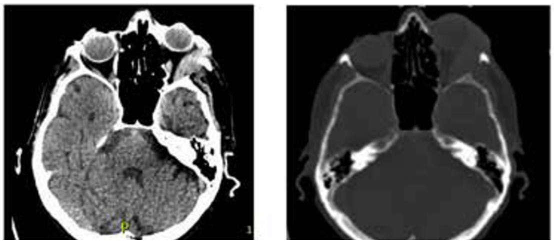

CT lacrimal mass
F88 presented with progressive diplopia and unilateral proptosis left eye. MRI was not possible due to claustrophobia. Non enhanced CT shows a spontaneously hyperdense mass at the location of the left lacrimal gland. Differential diagnosis: unilateral lymphoma, with adenoid cystic carcinoma and IOI being less likely. Pathology proved it to be a mantle cell lymphoma.

**Conclusion:** This poster provides a comprehensive review of the radiological aspects of anatomy and relevant pathologies of the lacrimal apparatus.
We will emphasize the potential role of multiparametric MRI techniques for characterizing lesions and focus on the clinical relevance for the ophthalmologist.

### Pathology of the nasolacrimal drainage pathway: Review of anatomy and imaging features

*Authors:* *S. Grauwels, P. de Graaf, M. De Win; Amsterdam University Medical Centers, Radiology & Nuclear medicine, Amsterdam, Netherlands*

**Learning objectives:**
- Anatomy of the nasolacrimal drainage pathway.
- Common pathologies of the nasolacrimal drainage pathway and their respective imaging features.
- Considerations of the ophthalmologists and other surgical colleagues.

**Background:** The anatomy of the nasolacrimal drainage pathway is an area many radiologists are unfamiliar with, and which may be overlooked or misinterpreted in a routine search pattern.
This is relatively small anatomic area, in which a wide array of pathologies may be present, some of which can be clinically diagnosed and evaluated. However, in some situations imaging can be necessary, allowing the radiologist to provide important information for referring physician and help guide further treatment and planning.

**Imaging findings and procedure details:** This poster shall primarily focus on the CT and MRI imaging characteristics of anatomy and different pathologies. This includes infection/inflammation, benign and malignant tumors.
We will also emphasize what the ophthalmologists/orbital and oculoplastic surgeons wants to know from us.

Example of the use of conventional and multiparametric MRI sequences:

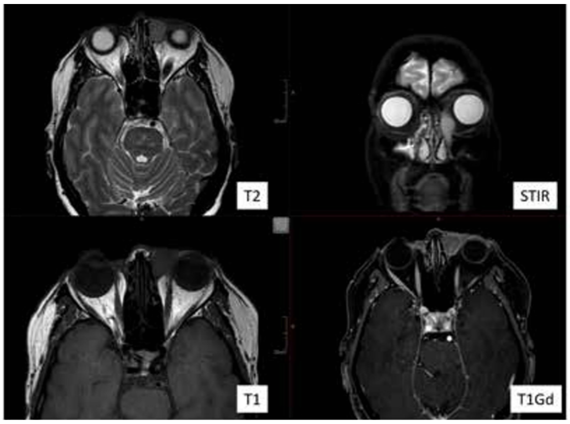

Example of multiparametric MRI, part 1
F61 presented with non-painful swelling in the area of the left lacrimal sac left since 6 months. Conventional MRI showed an enhancing mass in the area of the left nasolacrimal sac, extending into the nasolacrimal duct. With multiparametric sequences it was possible to narrow the differential diagnosis to a malignant lesion, most likely a lymphoma or a carcinoma. Pathology confirmed it to be a marginal zone lymphoma.

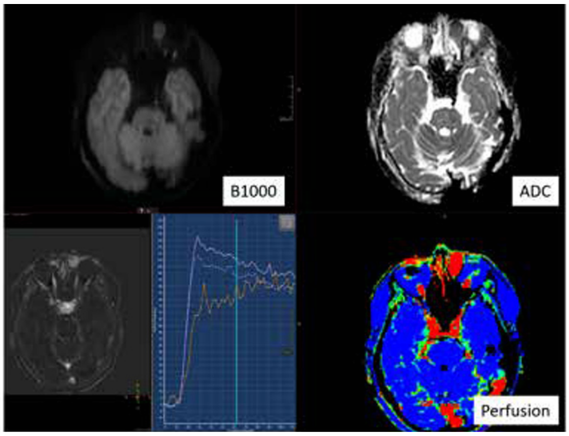

Example of multiparametric MRI, part 2
F61 presented with non-painful swelling in the area of the left lacrimal sac left since 6 months. Conventional MRI showed an enhancing mass in the area of the left nasolacrimal sac, extending into the nasolacrimal duct. With multiparametric sequences it was possible to narrow the differential diagnosis to a malignant lesion, most likely a lymphoma or a carcinoma. Pathology confirmed it to be a marginal zone lymphoma.

**Conclusion:** This poster provides a comprehensive review of the radiological aspects of anatomy and relevant pathologies of the nasolacrimal drainage pathway, as well as focus on the clinical relevance for the ophthalmologist.

### Pearls and pitfalls in diagnosing obstructive sinonasal disease.

*Authors:* *D. Du, G. Madani, K. Bhatia, O. Francies; Imperial College Healthcare NHS Trust, Department of Imaging, London, United Kingdom*

**Learning objectives:**
We will present a series of CT and MRI cases from our institution demonstrating the recognized patterns of sinonasal obstruction, including benign and malignant causes responsible for these. We describe some pearls and pitfalls in making a radiological diagnosis of sinonasal disease, and offer suggestions on how to avoid common misinterpretations.

**Background:** Obstructive sinonasal disease can be treated medically or surgically depending on pattern and extent of disease identified on CT/MR imaging. Management requires accurate radiological diagnosis as treatment of sinonasal disease is different depending on aetiology.

**Imaging findings and procedure details:** CT: Volumetric images: 1.5mm or 0.63mm slice width, with coronal and sagittal reconstructions.
MR: Multiplanar images: T1WI, T2WI, T1WI + contrast, +/- DWI.
A selection of cases follows. See poster for more cases and the final diagnoses.

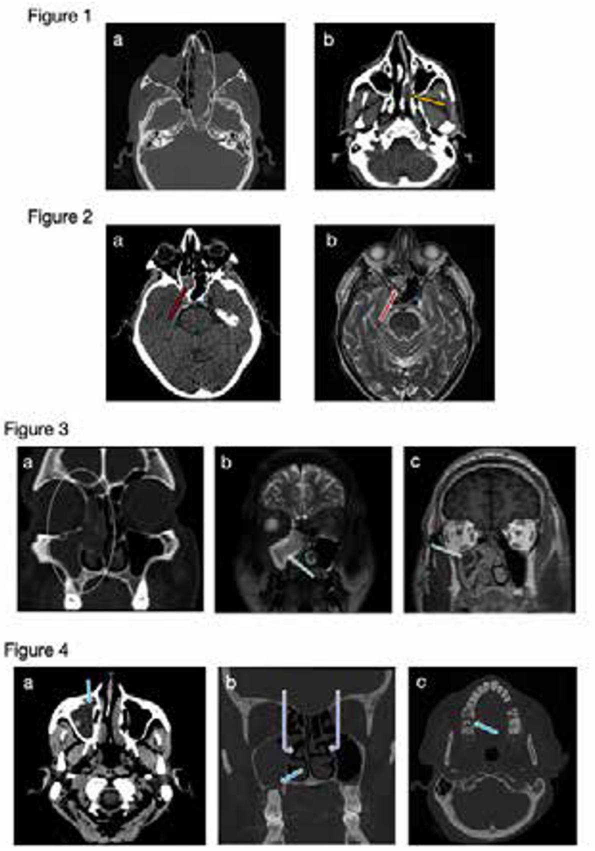

**Figure 1:** Sinonasal polyposis pattern **(a)** Axial CT (bone): Opacified left sphenoid and ethmoid sinuses, with left sphenoethmoidal recess obstruction. **(b)** Axial CT (soft tissue): Left choanal mucoid density mass extending into the ipsilateral maxillary sinus through a widened ostium.
**Figure 2:** Sphenoethmoidal recess pattern **(a)** Axial CT (soft tissue): Opacified right sphenoid sinus with hyperdense content protruding into sphenoethmoidal recess. **(b)** MR: Heterogenous signal with convoluted appearance filling the right sphenoid sinus on T2WI.
**Figure 3:** Ostiomeatal unit pattern **(a)** Coronal CT (bone): Opacification of the right maxillary sinus, frontal sinus, and anterior ethmoid air cells. **(b)** MR T2WI: Marked circumferential T2-weighted mucosal thickening, with expansion medially into the middle meatus and leftward nasal septal deviation. **(c)** MR T1WI + gadolinium: A mucous retention cyst is seen in the antrum postero-superiorly.
**Figure 4:** “Pitfall” **(a)** Axial CT (soft tissue), imaged for unrelated condition: Opacified right maxillary sinus with hyperdense material. Reported differential of fungal sinusitis **(b)** Coronal CT (bone): Odontogenic breach of right maxillary cortex. Note the patent middle meatusus. **(c)** Axial CT (bone): periapical lucency of upper right 7th molar.

**Conclusion:** It is important to recognize the patterns of obstruction in order to establish the true aetiology of obstructive sinonasal disease, as this will direct management. Appreciation of the various limitations of CT is key to avoiding common misinterpretations. Odontogenic disease is commonly overlooked and merits closer attention.

### Post-treatment imaging of the oral cavity tumors: Tips and tricks

*Authors: M. Fiannacca[1], S. Caprioli[2], F. Borda[1], N. Romano[3], A. Castaldi[3], G. Cittadini[4]; [1]University of Genoa, School Medicine of Radiology, Radiology, San Martino Hospital, Genoa, Italy, [2]University of Genoa, Radiology, Genoa, Italy, [3]Galliera Hospital, Neuroradiology, Genoa, Italy, [4]San Martino Hospital, Radiology, Genoa, Italy*

**Learning objectives:** To learn strength and limitation of contrast enhanced computed tomography (CECT), magnetic resonance imaging (MRI) and positron emission tomography (PET-CT) in treated oral cavity tumors.
To recognize expected post-surgical and post-radiotherapy changes.
To identify unexpected findings, as complications of treatment or persistent/recurrent disease.
**Background:** Head and neck cancer is the sixth most common type of cancer, and oral squamous cell carcinoma (OSCC) is the most common malignant tumor of the mouth.
Complete surgical resection is the standard of care of OSCC; advanced cancers are treated with a combination of surgery, radiotherapy and/or chemotherapy.
Familiarity with the expected post-therapeutic imaging appearance of the OSCC is crucial for individuating surgical complications and persistent or recurrent disease.
**Imaging findings and procedure details:** Expected post-surgical changes may be divided into destructive and reconstructive changes. Tissue loss, bone defect and neck dissection are included in the first category. Tissue transfers from local or distant sites used for close large defects are included in the second one (flaps or grafs). Two types of flaps can be described:
1. pedicle flaps, harvested either locally (local pedicle flaps) or regionally (regional pedicle flaps);
2. free flaps, that are transferred from a distant site and anastomized with local vessels. Post-radiotherapy changes vary over time and are characterized by inflammatory changes in the earlier phase and by fibrotic changes in late phase.

Unexpected changes may be due to recurrence or complications. Possible complications after surgery are: wound infection, fistula, flap necrosis, hematoma.
Post-radiotherapy complications include: osteoradionecrosis, radiation-induced lung disease, radiation-induced brain necrosis, radiation-induced tumors.
Imaging examinations of the neck after treatment are complex, with high inter-reader variability. NI-RADS is a standardized reporting template for CECT and PET/CECT that is designed to facilitate communication between radiologists and referring physicians with linked management recommendations.
**Conclusion:** Knowledge of the expected changes induced by therapies are essential for the correct interpretation of the posttreatment imaging of OSCC. A standardized approach may help a multidisciplinary team to correctly manage a suspected persistent or recurrent disease.

### US-Guided Percutaneous Microwave Ablation for Benign Thyroid Nodules – Snapshot of Lisbon Update

*Authors: L. Fernandes[1], A. Peixoto[2], F. Ribeiro[3], C. Guerreiro[4], S. Reimao[5], J. Sardinha[6], J. Leitao[2]; [1]Santa Maria Hospital- CHLN/CUF, Radiology, Lisbon, Portugal, [2]Santa Maria Hospital, Radiology, Lisbon, Portugal, [3]Faculty of Medicine, Imagiology, Lisbon, Portugal, [4]Santa Maria Hospital/CHLN, Radiology, Lisbon, Portugal, [5]Faculty of Medicine, Radiology, Lisbon, Portugal, [6]CUF Tejo Hospital, Radiology, Lisbon, Portugal*

**Learning objectives:**
The Authors started US-Guided Percutaneous Microwave ablation of benign thyroid nodules in Lisbon on March 2021. In the last ESHNR Meeting they presented an Educational Poster on this issue, and this year the purpose is to re-discuss this hot topic based on current evidence and to update the work than has been done so far.
**Background:** Benign thyroid nodules are extremely common in adult population, mainly asymptomatic and with no need for treatment. Nevertheless, some can cause pressure symptoms, be cosmetic annoying or related to autonomously functioning problems. In those instances, Surgery and/or Radioiodine are the gold standard treatment nowadays, but modern non-surgical minimally invasive options are becoming appealing alternatives. Microwave is one of the thermal ablation procedures available. It doesn´t need hospitalization, has lesser risks like recurrent laryngeal nerve palsy, result in minimal or even no scar, and doesn´t cause iatrogenic hypothyroidism.
**Imaging findings and procedure details:** The Authors sum up the state-of-the-art on the principles, basics procedure details and Imaging Findings of Microwave thyroid Ablation.
By the time of this Abstract, they treated 13 patients, with good results paralleling literature reports and no major complications. Based on their personal experience so far, many technical aspects will be extensively illustrated and debated, hopping to create a useful brainstorm on this issue.
**Conclusion:** There is an increasing interest in Microwave Ablation and is becoming gradually an important tool to treat several benign thyroid nodules. However, longer-term outcomes require further validation and is still recommended as a second-line modality in most instances. The Author's aim is to give a Snapshot of Lisbon Microwave Thyroid Ablation update results so far, to support its technically feasibility and good results paralleling the more conventional Radiofrequency approach as well as to gather further adepts of such a growing and undoubtedly promising technique.

### Variants and Pathology Mimics in the Untreated Skull Base, Face and Neck

*Authors: U. Nnajiuba, R. Srinivasan, S. Connor; Guys & St Thomas' NHS Foundation Trust, Radiology, London, United Kingdom*

**Learning objectives:** To be able to recognize anatomical variants and foreign bodies/devices in head and neck imaging that can mimic or be mistaken for pathology.
**Background:** Asymmetries and atypical appearances in the head and neck due to normal anatomical variants and foreign bodies/devices can mimic pathology. It is vital that radiologists can confidently recognize and dismiss these to prevent patients being subjected to unnecessary investigations and interventions that induce anxiety and present risk of morbidity.
**Imaging findings and procedure details:** We present a pictorial review of opportunistically curated head and neck cases to illustrate examples of variants, asymmetries, developmental defects and accessory structures related to lymphoid, muscular, glandular and vascular anatomy. We will also demonstrate how foreign bodies and artefacts may mimic pathology at skull base, upper aero digestive tract, face and neck soft tissue locations.
**Conclusion:** There are a variety of atypical appearances that can be encountered in head and neck images due to anatomical variants and foreign bodies that can be mistaken for pathology. An awareness of these is essential for the radiologist so as not to recommend unnecessary investigation, intervention and potentially harm to patients.

### Various faces of skull base meningioma

*Authors: M. Stankov[1, 2], D. Hadnadjev Simonji[1, 2], M. Jukovic[1, 2]; [1]University of Novi Sad, Faculty of Medicine, Novi Sad, Serbia, [2]Clinical Centre of Vojvodina, Centre for Radiology, Novi Sad, Serbia*

**Learning objectives:**
- to present different localization of skull base meningioma based on the reports from our institution;
- to explain the role of CT and MR imaging in assessment of skull base meningiomas;
- to familiarize the radiologists with mimics of skull base meningiomas.

**Background:** Meningiomas are meningothelial cell neoplasms arrising from the arachnoid and are usually attached to the inner surface of the dura matter. They are extra axial, have sharp contour and lentiform or sessile shape. Skull base meningiomas include paranasal or olfactory groove, optic sheath, sphenoid wing, suprasellar, clivus, foramen magnum, tentorial and posterior fossa localization. They account about 36–50 % of all meningiomas. Hyperostosis of the underlying bone is often seen in skull base meningioma. MRI provides superior contrast resolution, allowing precise characterization and differentiation between extra- and intra-axial lesions. Meningiomas are often asymptomatic but larger tumors can cause both indistinct symptoms such as headache and dizziness and site-specific symptoms due to invasion of local stuctures (anosmia, ophthalmoplegia, lower cranial nerve palsies). Differential diagnosis includes metastasis and multiple myeloma.
**Imaging findings and procedure details:** We provide CT and MR images of skull base meningioma with the emphasis on different localization and relationship with surrounding structures.
**Conclusion:** Wide range of pathology can occur in a skull base and radiology plays vital role in localization and characterization of these lesions. The presented typical features of skull base meningiomas are important for radiologists to be familiar with in order to make proper differential diagnosis and avoid mimcs. Furthermore, precise assessment of skull base meningioma and its relationship with surrounding structures is crucial due to possible surgery.

### What the otolaryngologist wants to hear from you? Reporting pathologies of parapharyngeal space in contex of surgical approach.

*Authors: A. Knurowska[1], J. Piatkowski[2], T. Nowicki[1]; [1]Medical University of Gdansk, 2nd Department of Radiology, Gdansk, Poland, [2]Medical University of Gdansk, Department of Otolaryngology, Gdansk, Poland*

**Learning objectives:**
1. To characterize imaging findings of parapharyngeal space lesions.
2. To present different surgical approaches used in the management of parapharyngeal space lesions and how radiology assessment can prevail in surgeon decision-making.

**Background:** Parapharyngeal space (PPS) tumours account for 0.5 % of all head and neck masses. They represent a formidable diagnostic and treatment challenge. Surgery remains the standard treatment for most cases.
**Imaging findings and procedure details:** Computed tomography (CT), magnetic resonance imaging (MRI) and angiography are used for evaluation of different types of PPS tumours. Apart from making the most propable diagnosis and building-up a differential diagnosis, it is important to include some critical information in radiological report for surgeons.
PPS neoplasms are either located in the prestyloid or retrostyloid compartment. The most common primary lesions in the prestyloid compartment are salivary gland tumours. In retrostyloid compartment there are mainly neurogenic lesions (i.e. Schwannomas and paragangliomas), which usually displace the ICA and the prestyloid fat.
Traditional, transcervical approach is still commonly used, yet the importance of transoral and transoral robotic surgery (TORS) is rising. Transoral excision can eliminate the risk of first bite syndrome and a neck incisional scar. However, it offers limited exposure and poor visualization of key structures. TORS candidates are patients with well circumscribed lesion which is located more than 10mm from the skull base and with lateral displacement of the ICA. If the tumour appears infiltrative, with possible involvement of the great vessels, or displaces the carotid medially, a transcervical approach should be considered.
**Conclusion:** The diagnostics of PPS tumours are challenging because of their low occurrence rate, anatomical complexity of the space, and histological diversity. A precise knowledge of the patient's anatomy is essential to tailor surgical plan.
